# Advancing EEG-based assessment of consciousness and cognition in prolonged disorders of consciousness

**DOI:** 10.1038/s43856-026-01574-x

**Published:** 2026-04-17

**Authors:** Naomi du Bois, Attila Korik, Stephanie Hodge, Leah Hudson, Ainjila S. Elahi, Alain Bigirimana, Natalie Dayan, Jose M. Sanchez-Bornot, Alison McCann, Kudret Yelden, Lloyd Bradley, Krishnan P. S. Nair, Simon Judge, Damon Hoad, Emma Vines, Venu Harilal, Sheryl Parke, Paul Johnson, Jacqueline Pogue, Emma Dodds, Abayomi Salawu, Raymond Carson, Karl McCreadie, Jacqueline Stow, Jacinta McElligott, Aine Carroll, Damien Coyle

**Affiliations:** 1 Bath Institute for the Augmented Human (IAH), University of Bath, Bath, UK; 2 Intelligent Systems Research Centre, Ulster University, Derry, UK; 3 Queens University Belfast, Belfast, UK; 4 National Rehabilitation Hospital, Dublin, Ireland; 5 Kings College Hospital, London, UK; 6 Royal Hospital for Neuro-disability, London, UK; 7 Sheffield Teaching Hospitals NHS Foundation Trust, Sheffield, UK; 8 Barnsley Hospital NHS Foundation Trust, Barnsley, UK; 9 South Warwickshire University NHS Foundation Trust, Rugby, UK; 10 Norfolk Community Health and Care NHS Trust, Norwich, UK; 11 Western Health and Social Care Trust, Derry, UK; 12 Northern Health and Social Care Trust, Antrim, UK; 13 Oxford University Hospitals NHS Foundation Trust, Oxford, UK; 14 Hull University Teaching Hospitals NHS Trust, Hull, UK; 15 Health Sciences Centre, University College Dublin, Dublin, Ireland

**Keywords:** Predictive markers, Computational neuroscience, Cognitive neuroscience

## Abstract

**Background:**

Accurate assessment of residual awareness in patients with Prolonged Disorders of Consciousness (PDoC) remains a major clinical challenge, as conventional behavioural tools can underestimate covert cognition. This study evaluates whether a structured, multi-phase motor imagery Brain–Computer Interface (MI-BCI) protocol provides objective electroencephalography (EEG)-based indicators of awareness that complement behavioural assessments.

**Methods:**

Forty-four participants (*N* = 44) completed repeated imagined-movement tasks using wearable EEG (PDoC: Unresponsive Wakefulness Syndrome (UWS, *n* = 14), Minimally Conscious State (MCS, *n* = 17), Locked-In Syndrome (LIS, *n* = 11); two able-bodied participants as benchmarks; ClinicalTrials.gov: NCT03827187; 30-01-2019). The protocol assessed sensorimotor rhythm modulation, training with and without neurofeedback, and binary question answering across phases. Standard behavioural assessments (CRS-R and WHIM) were administered at each session.

**Results:**

Significant MI-BCI decoding accuracy (DA) is achieved by 73.8% of patients, of whom 90% progress to Q&A testing and frequently exceed the 70% usability threshold, revealing marked inter-individual heterogeneity. For significant MI-BCI runs, LIS outperform MCS (*p* = 0.007) and UWS (*p* = 0.048), while UWS exceed MCS during Q&A (*p* = 0.049), driven by familiar-voice stimuli. Using leave-one-subject-out cross-validation, combining predictions from DA and behavioural assessments improves balanced diagnostic accuracy to 62% (from 55%), increasing sensitivity to MCS (39% to 69%), with a modest reduction in LIS sensitivity (78% to 67%). Task-related activity over sensorimotor and parietal cortices differentiate diagnostic groups.

**Conclusions:**

The structured MI-BCI protocol demonstrates potential as a movement-independent, EEG-based tool for distinguishing UWS, MCS and LIS. Integrating DA and spatial patterns yields diagnostic information that may augment behavioural assessment and advance objective tools for evaluating awareness in PDoC.

## Plain language summary

Some people with severe brain injuries seem unresponsive, yet their brains may still show signs of awareness. This study tested a brain-computer system that detects these signs by recording tiny electrical signals from the scalp when a person imagines moving. We worked with people in different states of consciousness and those with locked-in syndrome, a rare condition marked by complete paralysis, but intact consciousness – across hospitals, care homes, and private homes. The results showed that many could change their brain signals, suggesting intentional thinking, especially those with locked-in syndrome. These findings show that this kind of brain-computer system could help doctors detect hidden awareness in people who cannot move or speak, and may eventually assist with diagnosis and basic communication.

## Abstract (web summary)

Du Bois et al. use a structured, multiphase brain–computer interface to test how people with severe brain injuries can modulate brain activity through imagined movements. The results show that decoding these signals provides objective evidence of awareness and diagnostic information beyond traditional behavioural assessments.

## Introduction

Disorders of Consciousness (DoC) refer to a spectrum of altered conscious states resulting from an acquired brain injury. When a DoC persists beyond 4 weeks, it is termed a Prolonged Disorder of Consciousness (PDoC)^1^. Consciousness comprises two components: wakefulness, driven by subcortical arousal systems (primarily the brainstem and thalamus), and awareness, which depends on a distributed frontoparietal network supporting higher-order cognitive processing and perception of self and environment^2–4^. Clinical presentations are on a continuum: coma (no wakefulness or awareness, typically transient); Unresponsive Wakefulness Syndrome (UWS), wakefulness is present, but awareness is absent; and the Minimally Conscious State (MCS), wakeful with minimal or fluctuating awareness^1^. In the UK alone, it is estimated that there are between 4000 and 16,000 patients diagnosed with UWS, with up to three times as many living in MCS^5^. Although not part of the DoC spectrum, Locked-In Syndrome (LIS) can be misdiagnosed as a DoC using behavioural assessment tools, as the condition results in near-total paralysis that can mask preserved awareness^6–8^. People with LIS retain consciousness awareness and can typically communicate via eye movements or blinking, although this ability is lost in Complete Locked-In Syndrome (CLIS) due to additional oculomotor impairment^7,8^.

Several diagnostic tools are available to assess consciousness, including the Coma Recovery Scale-Revised (CRS-R), the Wessex Head Injury Matrix (WHIM), and the Sensory Modality Assessment and Rehabilitation Technique (SMART)^9^. However, a fundamental limitation shared by all behavioural assessment tools is their reliance on the patient’s ability to produce overt motor or verbal responses. For patients with a PDoC, this presents a significant challenge such as fluctuations in arousal, sedative side effects of medication, the presence of a tracheotomy, or severe motor impairments may prevent consistent behavioural output. Assessment quality is also influenced by clinician expertise and experience^10^. Consequently, up to 40% of MCS patients are misdiagnosed as being in UWS^10,11^. Accurate diagnosis is critical for appropriate prognostication, to maximise quality of life and to guide decisions around longer-term continuation of treatment^10–13^. Misdiagnosis is not limited to PDoC. A survey of forty-four LIS patients in France reported an average diagnostic delay of 78.1 days, with some cases exceeding 1460 days (4 years), attributed to misinterpretation of symptoms^6^. These findings highlight the urgent need for objective diagnostic tools that do not rely on motor behaviour or vocalisation alone to assess consciousness.

Alternative approaches to behavioural assessment have emerged following advances in neuroimaging techniques. A landmark study by Owen et al. demonstrated covert awareness in a patient with an apparent UWS by measuring neural responses to motor imagery (MI) tasks using functional magnetic resonance imaging (fMRI)^14^. Monti et al. extended this fMRI-based protocol to achieve yes/no communication^14,15^. However, fMRI is costly and not always accessible, prompting the development of alternative methods such as electroencephalography (EEG). Cruse et al.^16^ demonstrated that EEG, a non-invasive, bedside-compatible method, could detect covert awareness in DoC. Sensorimotor rhythms (SMRs), typically in the 8–12 Hz (*µ*) and 18–26 Hz (*β*) ranges, are modulated during both movement and kinaesthetic motor imagery (MI)^17^. Cruse et al. demonstrated intentional SMR modulation in three UWS patients, indicating misdiagnosis or state transition^16^. Coyle et al.^18^ moved beyond one-off detection to closed-loop training for MCS patients, using a motor imagery brain–computer interface (MI-BCI), marking a shift toward BCI as a trainable skill. Participants received both auditory and visual real-time feedback across multiple sessions, with auditory feedback (particularly music) shown to improve motor imagery Decoding Accuracy (DA). This study demonstrated the feasibility of MI-BCI training in PDoC populations, catalysing a transition in EEG-based BCI research from passive testing to interactive, real-time systems for PDoC populations^19^.

Despite growing support for BCIs in supplementing the CRS-R, the current gold-standard behavioural assessment tool^9,20,21^, the Royal College of Physicians (RCP) guidelines continue to recommend behavioural assessments (favouring the CRS-R)^22^. The RCP argues that there is not enough evidence for neuroimaging-based methodologies to enter routine clinical practice alone^22^. In contrast, the American Academy of Neurology and the European Academy of Neurology recognise the additive potential of neuroimaging and electrophysiological tools under specific conditions^23,24^. All three organisations share concerns, however, regarding standardisation, cost-effectiveness, and real-world feasibility of these technologies^23^. Therefore, ongoing research continues to address these concerns by developing practical, scalable, and clinically integrated solutions.

Recent efforts have aimed to broaden the diagnostic capabilities of EEG-based BCIs in (P)DoC. In conjunction with, and subsequent to the Coyle et al.^18^ MI-BCI study, hybrid EEG-based BCI paradigms have been developed that combine visually evoked responses, i.e. the P300 event-related potential and the Steady-State Visually Evoked Potential to detect command following and preserved reasoning in this population^25,26^. This multi-input approach has improved BCI sensitivity and reliability, particularly when supported by advanced classification algorithms^18,27–31^. Kim et al.^32^ demonstrated the value of a multi-EEG marker approach to the assessment of cognitive function in a brain-injured paediatric cohort. By combining the EEG correlates of motor command-following and auditory evoked responses, they enabled cognitive profiling in this cohort, reinforcing the diagnostic/prognostic potential for this approach in clinical populations, highlighting the diagnostic potential of integrating multiple neural signals.

However, despite these advances, EEG-based MI-BCI systems remain blind to the integrity of the neural networks supporting consciousness. Functional connectivity (FC) analysis offers a systems-level view of the network dynamics that support awareness, including the Default Mode Network (DMN) and the Fronto-Parietal Network (FPN). Research findings show that disruptions in these intrinsic networks are consistently associated with loss of awareness^33–38^, while residual or reorganised connectivity in key hubs such as the medial Prefrontal Cortex (mPFC), Posterior Cingulate Cortex (PCC), and dorsolateral Prefrontal Cortex (dlPFC) can distinguish between DoC states^39,40^. Furthermore, cortico-thalamic integrity, particularly within the ascending arousal system, is an established neural marker for poor outcomes in acute DoC patients^41,42^. Combining FC with EEG-based MI-BCI could move diagnostic tools beyond binary classifiers toward network-informed diagnostics.

Building on this foundational work (e.g. Owen et al.^14^; Cruse et al.^16^; Coyle et al.^18^), the present study evaluates a multi-phase, longitudinal EEG-based MI-BCI protocol for patients with PDoC and LIS. The study protocol incorporates command-following assessment, real-time neurofeedback, and structured yes/no response evaluation to closed questions, rolled out in three phases: assessment (sessions 1–2), training and feedback (sessions 3–6), and binary question-answering (sessions 7–10/13). Phase I identifies participants capable of SMR modulation. Phase II provides guided MI practice with auditory feedback to support learning. Phase III investigates participants’ binary responses to closed biographical, numerical, logical, and situational questions using the MI-BCI. The primary focus is to determine whether cognition can be profiled by analysing the aggregate decoding performance within each question category, thereby using the BCI as a scientific instrument to probe domain-specific awareness. Importantly, the outcomes of this phase also provide an indication of the system’s potential for future development as a communication mechanism.

In addition to EEG-signal classification, used for group-level comparisons of DA, the study incorporates further EEG analyses to probe underlying neural mechanisms of awareness. Specifically, a between-group comparison of the features selected for classification is included, to provide insight into differential neural activation across diagnostic categories, as well as a between-group analysis of FC, targeting intrinsic networks implicated in awareness, to explore systems-level differences in network integrity between PDoC and LIS participants.

This study represents one of the largest EEG-based MI-BCI investigations to date involving patients with UWS, MCS, and LIS/CLIS (*n* = 42). Participants were tested in real-world environments, such as hospitals, care homes, and private residences, enhancing the system’s ecological validity and clinical relevance. Importantly, this work moves beyond one-off detection paradigms by integrating training, communication, and behavioural comparison (CRS-R, WHIM) in a longitudinal framework. The primary objective is to determine whether the structured BCI protocol provides an objective, scalable complement to existing diagnostic frameworks for patients with PDoC. Specifically, we assess whether DA from a two-class imagined-movement task differentiates diagnostic groups (UWS, MCS, and LIS) and reveals consistent neural markers of intentional modulation. In line with the study’s basic-science and diagnostic focus, the final phase, involving a structured closed Q&A, assesses the feasibility of extending this framework to future applications in understanding cognition in unresponsive patients, and explores whether responses may provide insights into patients’ cognitive state or needs.

We demonstrate that most participants achieve significantly above chance level MI-BCI DA, with LIS generally achieving higher accuracies than UWS and MCS across paradigms. Group-level modelling identifies DA as a key predictor distinguishing LIS from UWS, while the inclusion of traditional behavioural measures further improves classification. Despite paradigm-dependent variation in DA, overall DA, motor-associative cortical activation patterns, and connectivity features supplement existing behavioural indicators, suggesting that DA offers complementary diagnostic information beyond traditional behavioural assessment. Exploratory analyses using domain-specific question stratification (i.e. grouping yes/no items by cognitive category) revealed promising initial evidence that supports further development of refined BCI-based assessment tools capable of probing distinct cognitive domains and, potentially, enabling more effective communication within this framework.

## Methods

### Study sample

This study was registered on ClinicalTrials.gov (NCT03827187; 30th January, 2019), and included 42 patients (aged 17–73 years; 13 females, 29 males). Patients were diagnosed as follows: 14 with UWS, 17 with MCS, 10 with LIS, and 1 with CLIS (total LIS participants, *n* = 11). Two able-bodied males (aged 20 and 23) were included as benchmarks (AB, *n* = 2). Previous publications involving subsets of this cohort have reported early findings^18,43,44^, with a recent preprint reporting on the findings of a separate evaluation^45^. All participants were naïve to the task, with the exception of one MCS participant who had previously taken part in an earlier study by Coyle et al.^18^ that investigated the ability of MCS patients to learn to modulate SMRs through MI-BCI training.

Ethical approval was granted by the NHS Health Research Authority (Integrated Research Application System UK and Scotland, Project IDs: 136640 and 247815; Research Ethics Committee (REC) reference: 18/WA/0186; approved 07/10/2020). Ethical approval to conduct this research study in the Republic of Ireland, was granted by the National Rehabilitation University Hospital (NRH) REC. Informed consent was obtained from participants or via proxy consent where necessary. The approved protocol involved experimental procedures that did not include any therapeutic or clinical intervention; accordingly, the research was conducted as a non-interventional basic science study. All procedures complied with the Declaration of Helsinki.

Clinical consultants screened patients for eligibility and collected consent. For participants lacking capacity, consent was provided by a consultee, such as the participant’s next of kin or legally authorised carer. Inclusion criteria were a diagnosis of PDOC, low awareness states, or (C)LIS. Exclusion criteria included progressive neurological diseases, uncontrolled epilepsy or pain, medications impairing cognition or causing excessive fatigue, and skull deformities preventing electrode contact. Sex and gender were not variables under investigation; however, participants’ sex is reported in the population description. The study population did not include socially relevant subgroupings; therefore, race and ethnicity were not reported.

### Experimental paradigm

Movement imagery tasks were selected for each patient with input from family or clinicians, avoiding injured brain regions and favouring arm movements when possible. Patients imagined one movement combination throughout the study: left vs. right arm, right arm vs. feet, or left arm vs. feet. Before each session, patients were instructed to keep their eyes open, remain still, and avoid teeth grinding. With consent from a family member or caregiver, a gentle massage between runs was permitted to rouse the patient if they appeared to fall asleep.

The study consisted of three phases (see Table 1).

**Table 1:** Structure of the study phases and associated runs

	Run	1	2	3	4
Phase I (Assessment paradigm)	Session 1	A1	F(PN)	-	-
	Session 2	A2	T	F(PN)	-
Phase II (Training and Feedback paradigms)	Sessions 3–4	T	T	F(PN)	F(M)
	Sessions 5–6	T	F(M)	F(M)	F(M)
Phase III (Q&A paradigm)	Sessions 7–8	T	F(M)	Q&A	Q&A
	Sessions 9–10	F(M)	F(M)	Q&A	Q&A
	Sessions 11–12	F(M)	Q&A	Q&A	Q&A

For some participants, training, feedback and Q&A sequencing was adjusted to accommodate individual progression and responses.

*A* Assessment, *F(PN)* Feedback (Pink Noise), *T* Training, *F(M)* Feedback (Music), *Q&A* Question and Answer session.

#### Phase I (Sessions 1–2)

*Assessment* of awareness through imagery tasks following cues, plus real-time Feedback runs. Data were analysed offline (see Offline single-run analysis for BCI calibration for details). Participants advanced to the next phase when DA exceeded 70% or when task-related DAs were statistically greater than baseline (one-tailed paired *t-*test, *α* = 0.05). The 70% threshold corresponds to performance well above the 99% upper bound of the binomial chance level for 90 trials, providing a conservative operational benchmark for reliable BCI control.

#### Phase II (Sessions 3–6)

Additional Training and Feedback runs.

#### Phase III (Sessions 7–10)

Training, Feedback, and Q&A runs.

**Fig. 1:**
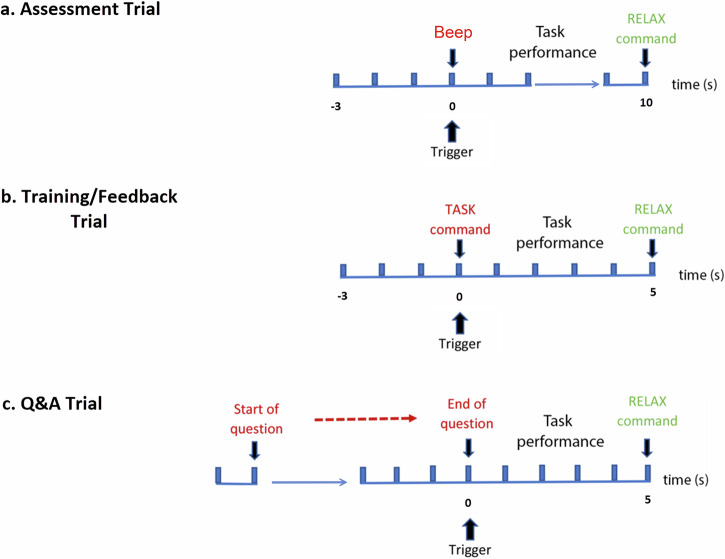
Timing of the trials. **a** Assessment. **b** Training, and Feedback. **c** Q&A. Note: the Q&A task began with a question. Therefore, the trigger for the start of the task performance period was sent at the end of the question. Paradigm-specific trial instructions are provided in Supporting Information, Supplementary Note 1.

Trial timings are presented in Fig. 1. Participants who advanced completed 4–13 sessions, each comprising 1–5 runs, although the number varied depending on health and scheduling. Tasks were performed without distractions or visual aids once sessions began.

### Experimental procedure

Each daily session (1–2 h) included 1–5 runs selected from Assessment, Training, Feedback, and Q&A paradigms. EEG setup was performed at the start of each session. The verbal instructions given (via headphones) at the start of each task are provided in Supporting Information: Methods, Supplementary Note 1. Paradigm Specific Instructions.

#### Assessment runs

Patients were verbally and visually instructed to imagine specified movements (e.g. lifting a weight with the right arm). Each run followed a block design: six blocks of 15 trials, alternating between two imagined movements (three blocks per movement). Beeps (0.5 s) cued imagery every 8 s, with pre-recorded instructions at block start. Each Assessment run lasted ~17 min, including rest periods. The protocol was adapted from Cruse et al.^16^.

#### Training runs

Conducted at the start of each Phase II/III session, Training runs compressed the Assessment format: 60 randomly ordered trials (30 per movement class) without block structure. Commands (‘Left’/‘Right’) were given via pre-recorded voice, followed by a 5 s imagery period and 3 s relax cue. Each Training run lasted ~8 min.

#### Feedback runs

Similar to Training runs, but included real-time auditory feedback. Sound (broadband noise or music) shifted azimuthally between ±90° based on the correct decoding of imagined movements. Misclassification led to erratic or misdirected auditory feedback. Runs lasted ~8 min.

#### Q&A runs

Each Q&A run (8–11 min) presented 48 yes/no questions from two combined categories (basic logic, numbers/letters, situational, biographical) using familiar caregiver-recorded audio to produce self-relevant stimuli which were expected to maximise engagement^46,47^. Patients responded by imagining movements associated with ‘yes’ or ‘no’. Task periods lasted 5 s, followed by relax cues. Categories rotated to balance linguistic and numerical content. Questions were adapted from the Montreal Cognitive Assessment^48^ and in accordance with the National Clinical Guidelines for PDoC^49^. A full list of the questions for each category is provided in Supporting Information: Methods, Supplementary Note 2. Questions were personalised by inserting the correct (for a “yes” response) or incorrect (for a “no” response) question completion information where there are blanks in the text. For example, the biographical question set contains the question “Is your name _________?”. This question is asked twice in one biographical question run—once with the participant’s correct name as a “yes” response question, and once with the participant’s incorrect name as a ‘no’ response. Questions were presented in random order.

#### Clinical measures

Routine CRS-R and WHIM assessments^50–52^ were conducted daily to compare behavioural scores with EEG-based BCI performance (i.e. DA). Sessions alternated between morning and afternoon to account for diurnal variation in patient alertness^53^.

#### Data acquisition

**Fig. 2:**
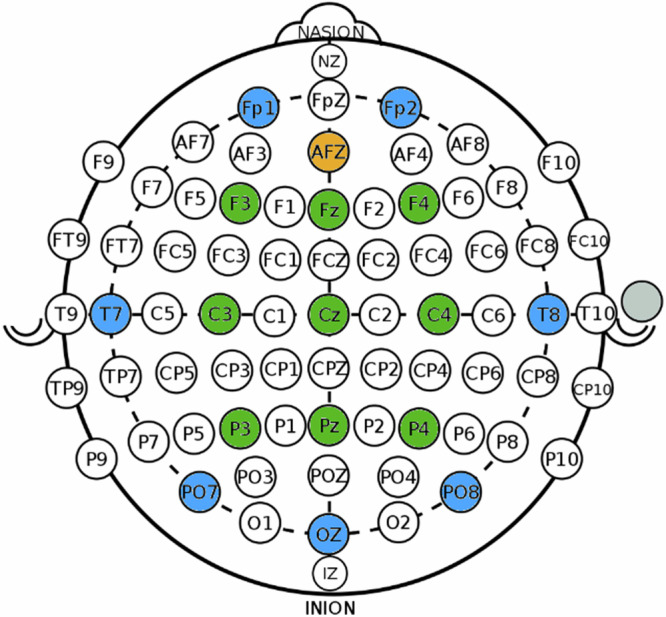
EEG montage. The positions of the EEG (blue and green) and ground (orange) electrodes are presented in this figure. Nine EEG channels covering motor and imagined movement-related cortical areas are indicated in green. The reference clip was attached to the right ear (as indicated by the circle in grey).

EEG was recorded from 16 channels (Fig. 2) with active electrodes using a g.Nautilus Wireless Research EEG headset^54^. The reference electrode was fixed on the right earlobe, and the ground electrode was positioned over the AFz electrode location according to the international 10/20 EEG standard. The EEG was filtered (Butterworth, 0.5–100 Hz, eighth order), and sampled (sampling rate: 250 Hz, down-sampled to 125 Hz).

A user datagram protocol managed communication between a MATLAB Simulink^55^ module (that was used for EEG data acquisition and online signal processing), and the experimental protocol controller application presented in the Unity 3D Game Engine^56^.

### Offline signal processing

**Fig. 3:**
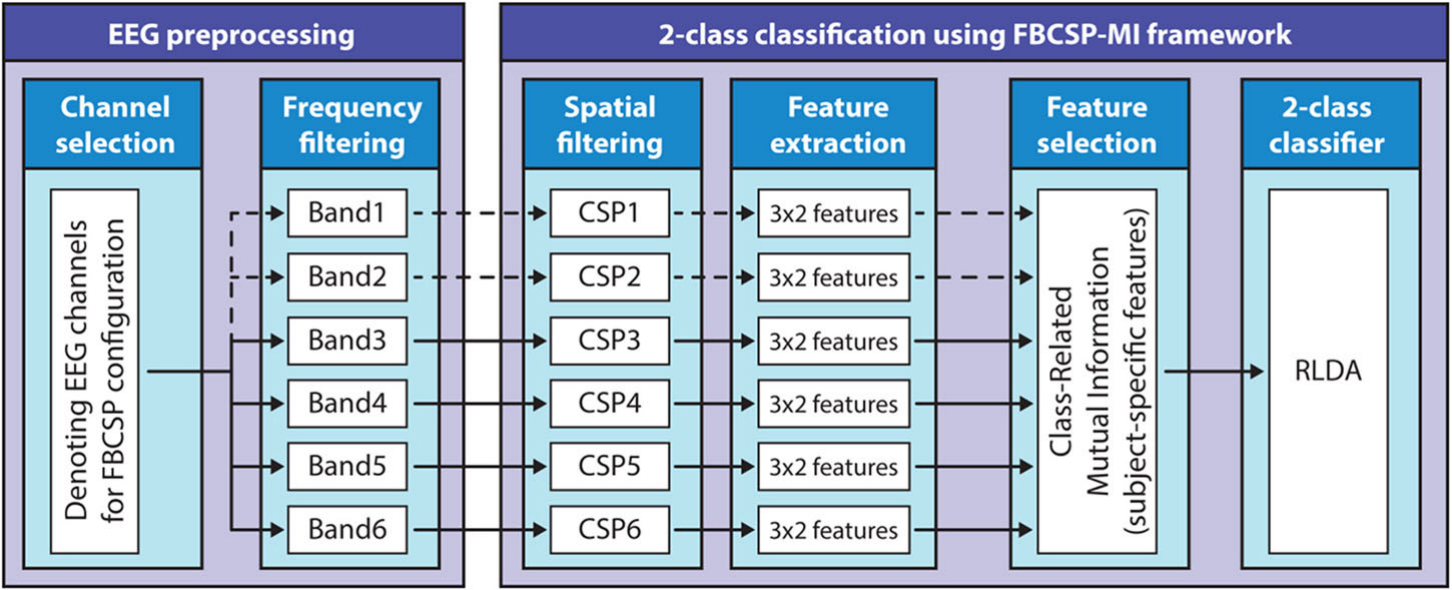
Structure of the applied FBCSP-MI-based 2-class classification method. The block diagram illustrates the signal processing framework, including filter bank common spatial patterns and mutual information (FBCSP-MuI) based features selection modules, and a regularised linear discriminant analysis (RLDA) 2-class classifier. The analysis was performed using three different sets of frequency bands, involving four adjacent bands from the six pre-processed bands (i.e. delta, theta, mu, low-beta, high-beta, low-gamma bands). The illustrated setup represents the highest four-band option.

Offline analysis and BCI calibration were performed using a filter-bank common spatial patterns (FBCSP) and mutual information (MuI)-based feature selection framework^57,58^ following a structure similar to Korik et al.^59^. This calibrated model was used to provide auditory feedback in online runs. FBCSP is a well-established EEG classification method for distinguishing between imagined movements^60,61^, as shown in Fig. 3.

EEG signals were filtered into six standard frequency bands [0.5–4 Hz (delta), 4–8 Hz (theta), 8–12 Hz (mu), 12–18 Hz (low beta), 18–28 Hz (high beta), 28–40 Hz (low gamma)] using Simulink FIR filters^55^ (band-pass attenuation: 0 dB; band-stop: 60 dB). For each participant and run, FBCSP-MuI calibration involved testing combinations of:

1. Two EEG channel sets [16-channel full coverage; 9-channel motor cortex-focused]
2. Three frequency band sets ([delta–low beta], [theta–high beta], [mu–low gamma], each comprising four adjacent bands from the six pre-processed bands, see Fig. 3)
3. Classification windows of 1 s and 2 s
4. Between 4 and 10 of the highest-ranked MI-selected features

### Feature extraction and classification

#### Epoching

For each run, task-relevant EEG segments were extracted from −3 s to +5 s relative to the onset of the motor imagery cue. These epochs were extracted from the frequency-filtered dataset and processed separately for each EEG channel configuration.

#### Spatial filtering (CSP)

A common spatial pattern (CSP) method was applied to maximise discriminability between two motor imagery classes. CSP generates a set of spatial filters that maximise the variance of EEG signals from one class while minimising it for the other^62,63^. Each filter corresponds to a row in a linear transformation matrix applied to the band-pass filtered EEG signals. For each frequency band, three CSP filter pairs (six filters total) were retained. These were selected based on their ability to produce the highest variance separation between classes.

#### Feature extraction

CSP-filtered signals were transformed into feature vectors using a sliding window of either 1 s or 2 s (depending on the FBCSP-MuI setup), with a 40 ms step size between windows. For each window, the feature vector was calculated as the natural logarithm of the signal variance:

1 $$\bar{\omega }=log ({var}(E))$$

where,$\,\bar{{{\boldsymbol{\omega }}}}$ is the feature vector and ***E*** is the CSP-transformed EEG signal.

#### Feature selection (mutual information)

Features were ranked and selected using a “best individual feature” algorithm based on mutual information (MuI)^58^. The MI between each feature and the class label was computed using a fixed quantisation level of 3. For each configuration, 4–10 features with the highest MuI values were retained for classification (evaluated across multiple configurations).

#### Classification (RLDA)

Selected features were classified using regularised linear discriminant analysis (RLDA), implemented via the RCSP toolbox^63^. RLDA constructs a linear decision boundary (hyperplane) that separates the two classes. Classification output is determined by the sign of the feature vector’s projection onto the weight vector:

- Positive output → Class 1
- Negative output → Class 2

The classifier’s decision confidence is proportional to the distance from the hyperplane^64^.

#### MI-BCI performance evaluation

DA values were computed for each setup (1 s vs. 2 s window, channel configuration, frequency band set, and number of features). These were compared across configurations to 1. determine the optimal BCI calibration per participant and 2. to maximise sensitivity at the individual level to detect evidence of intentional sensorimotor modulation within a BCI framework.

### Offline single-run analysis for BCI calibration

Each combination of FBCSP-MI setup parameters (channel set, frequency band set, classification window length, and number of features) was evaluated independently for every participant and run. Classification performance was assessed using six-fold cross-validation (CV). The configuration yielding the highest peak DA in the task period was selected as optimal for that participant and run (see Feature extraction and classification).

#### Time-varying decoding accuracy calculation

For each fold, DA was calculated at each time point, generating a time-resolved accuracy curve. The baseline (reference) interval was defined from −1000 to 0 ms before the task cue. Although motor imagery tasks were cued at 0 ms and nominally expected between 0 and 5000 ms, the task interval was defined from 400 ms to 7000 ms post-cue. This adjusted window accounts for potential stimulus-related activity in the first 400 ms (which could confound motor imagery signals), and known latency in PDoC patient responses, which may extend beyond 5 s post-cue^65^. A 200 ms moving average smoothing was applied to each DA time-series to reduce short-term variance.

#### Peak DA extraction

For both reference and task intervals, the smoothed DA curve was scanned to identify the maximum DA value (smoothed peak). A final peak DA was then selected from the non-smoothed DA curve as the local maximum within a ± 150 ms (300 ms total) window centred on the smoothed peak. This two-step process reduced the likelihood of selecting spurious DA spikes in otherwise low-accuracy regions.

#### Statistical comparison of task vs. baseline DAs

Task and baseline DAs were compared using a one-tailed paired *t*-test across CV folds, to test the hypothesis that DA during the task interval was higher than baseline. A statistically significant result at *α* = 0.05 indicated that classifier performance during motor imagery exceeded baseline variability. Runs without significant task-related increases indicated non-engaged runs. Following initial group analyses that focused specifically on differences in DA, subsequent analyses were conducted only on runs with significant DA. This procedure ensures that significant results reflect intentional modulation rather than random variability.

#### Evaluation against chance

Although the theoretical chance level for two-class classification is 50%, actual chance levels can be influenced by trial count, label distribution, and temporal correlations^66^. To assess whether observed DA exceeded chance performance, a permutation test was conducted:

- 100 permutations of the trial labels (class 1 vs. class 2) were generated.
- For each permutation, the full six-fold CV procedure was repeated, producing 100 time-resolved random DA curves.
- At each time point, the empirical *p* value was calculated as:

2 $$p=\frac{\left|\left\{{D}^{{\prime} }\in \hat{D}\,:{ac}\left(D{\prime} \right)\ge {ac}\left(D\right)\right\}\right|+1}{n+1}$$

where $\hat{D}$ is a set of *n*-randomised versions ${D}^{{\prime} }$ of the original data $D$, and ${ac}\left(D\right)$ is the accuracy achieved with the non-randomised data^67^. The null hypothesis (that observed DA could be obtained by chance) was rejected for *α* = 0.05. Time-resolved DA curves from both the original data and the permutation baseline were plotted together for visual comparison.

#### Paradigm-wise performance tracking

Peak DA values from each experimental paradigm (Assessment, Training, Feedback, Q&A) were extracted for each participant and session. These were visualised in separate plots to monitor consistency across paradigms.

### Frequency and topographical contribution analysis

To explore which frequency bands and EEG channels contributed most to class separation, the CSP filter outputs and associated mutual information (MI) weights were analysed post-calibration.

#### Band-wise contribution (heatmap basis)

At each time point *t*, the contribution of frequency band *b* to classification was computed as the average MuI weight of all features ${n}_{b}$ within that band:

3 $${M}_{b.t}=\frac{{\sum }_{{n}_{b}}{M}_{b,t}^{{n}_{b}}}{{N}_{b}}$$

where ${M}_{b,t}^{{n}_{b}}$ is the MuI weight of feature ${n}_{b}$ at time *t*, and ${N}_{b}$ is the number of features from band *b*.

#### Topographical contribution per band

To generate spatial maps of DA-relevant EEG activity, CSP filter outputs were weighted by their MuI scores:

4 $${F}_{{b,n}_{b},i,t}={C}_{i,t}^{{b,n}_{b}}{M}_{t}^{{b,n}_{b}}$$

where ${F}_{b,i,t}$ is the cumulative contribution of EEG channel *i* within frequency band *b* at time *t*. This is computed by summing over all features ${n}_{b}$ from band *b*, where each feature’s contribution ${F}_{b,i,t}^{{n}_{b}}\,$ is the product of the CSP transformation value ${C}_{i,t}^{{b,n}_{b}}$ for channel *i*, and its mutual information weight ${M}_{t}^{{b,n}_{b}}$. Thus, Eq. 1 aggregates these weighted contributions:

5 $${F}_{b,i,t}=\,{\sum }_{{n}_{b}}{F}_{b,i,t}^{{n}_{b}}\,$$

#### Multi-band channel contribution

To integrate across all frequency bands, a composite score for each channel was computed as:

6 $${F}_{i,t}=\,\frac{{\sum }_{b}{F}_{i,t}^{b}\,}{N}$$

where *N* is the number of bands.

#### Task vs. baseline topographical difference

Finally, to isolate spatial patterns specifically related to motor imagery (vs. baseline activity), the difference between multi-band contributions at peak task and baseline times was computed:

7 $${F}_{i,{t}_{1}-{t}_{0}}={F}_{i,\,{t}_{1}}-{F}_{i,{t}_{0}}$$

where, ${F}_{i,{t}_{1}-{t}_{0}}$ represents the difference in multi-band contributions for EEG channel *i* between the task period (${F}_{i,\,{t}_{1}}$) and the reference period (${F}_{i,{t}_{0}}$).

The topographical maps using mutual information weighted CSP filters are calculated at the sensor level using MATLAB^68^, and the location of source activity is estimated and plotted using the sLORETA software package^69^.

### Online BCI configuration

In online runs, real-time auditory feedback was generated using the optimised FBCSP-MuI configuration selected during offline calibration (see previous sections). For each time point *t* within a trial *n*, the classifier produced a time-varying signed distance (TSD), a scalar value representing the projection of the feature vector onto the RLDA decision boundary^70,71^. The TSD value was computed as described in 8:

8 $${{\mathrm{TSD}}}_{t}^{n}={w}^{T}{\bar{\omega }}^{n}-{a}_{0}$$

where, ${w}^{T}$ is the transposed weight vector (slope of the RLDA hyperplane), ${\bar{\omega }}^{n}$ is the feature vector at time *t* in trial *n*, and ${a}_{0}$ is the bias term (intercept of the RLDA hyperplane).

#### Interpretation of TSD

The sign of the TSD determines the predicted class (e.g. left vs. right imagery), and the magnitude of the TSD reflects the classifier confidence, which was mapped to the extent of auditory feedback movement (e.g. how far the sound panned toward the left or right ear)^18,72^.

#### Bias correction

To reduce systematic classification bias and improve feedback stability, the TSD was de-biased online by subtracting a rolling average of TSD values over the preceding 35 s. This dynamic correction helped stabilise the feedback direction over time, particularly in cases of class imbalance or drift.

### Offline analysis of Q&A category results

To explore the feasibility of the SMR-BCI system as a communication tool for patients with PDoC, offline analysis examined whether DA varied as a function of participant group, question category, and experimental paradigm. The groups included patients diagnosed with UWS, MCS, LIS, and AB controls. The four question categories consisted of biographical, numerical, logical, and situational content. Experimental paradigms included assessment, training, feedback, and Q&A runs.

The analysis replicated the methods described in the Offline Single-Run Analysis for BCI Calibration, applying the same procedures for feature extraction, classification, cross-validation, and peak DA calculation. For Q&A runs specifically, trials were grouped by question category, and DA was computed separately for each participant and group. These results were then compared across groups and categories to identify patterns in BCI communication performance. Additionally, all Q&A trials across the four categories were pooled into a general ‘question category’ to assess overall decoding performance within each participant group. An exploratory analysis was performed to evaluate the question-level accuracies. For each question category (Biographical, Situational, Basic Logic, Numbers/Letters) and participant group (UWS, MCS, LIS, AB), we computed confusion matrices comparing target to decoded class, using smoothed peak DA as the decision metric in the task and reference periods separately. From these matrices, we derived overall question-level accuracy, per-class bias indices, and per-class false-negative and miss rates.

### Statistics and reproducibility

All statistical analyses were performed using R-studio (version 4.4.2)^73^. Two-tailed tests were used unless otherwise specified, with a 95% confidence interval (CI) applied throughout. Statistical results are reported using exact *p* values where available; adjusted post-hoc comparisons are labelled and presented using standard threshold notation. The Shapiro–Wilk test is used to assess normality, and Levene’s test evaluates the homogeneity of variances across groups. Prior to hypothesis testing, data are screened for outliers using the interquartile range method. Outliers are retained unless they clearly influence the variance or distribution of the data, as such cases may reflect clinically meaningful variability rather than artefactual noise, given the heterogeneity of PDoC populations. To account for potential influence from these values, robust statistical approaches are employed. Specifically, for analyses of variance, Welch’s ANOVA is applied to mean DA data when assumptions of normality are met, as it is robust to unequal variances and sample sizes. The Kruskal–Wallis test is applied to median DA data, as it is appropriate for non-normally distributed data that may include meaningful extreme values. To control for Type I error across pairwise comparisons, where omnibus effects are significant, Games-Howell post-hoc tests are conducted for Welch’s ANOVA, and Dunn’s post-hoc tests with Holm correction are performed for the Kruskal–Wallis analyses. In addition, planned Welch contrasts are included to test a priori hypotheses comparing (1) UWS against the combined mean of MCS and LIS, and (2) MCS against LIS. In each case, sample size (including *n* per group), means, and standard deviations are reported descriptively in the Supporting Information, Supplementary Table 1a, b. These tables include the AB group as a benchmark. While not included in the inferential analyses, descriptive data for the AB group are provided to illustrate the performance of all diagnostic groups relative to healthy individuals. For further visualisation, the AB group is included in Figs. 5, 6 and 9. For all analyses, unless otherwise specified, only significant runs were used, i.e. motor imagery (MI)-BCI runs where peak DA during the task period was significantly higher than the corresponding peak baseline DA (one-tailed *t*-test, α = 0.05; consistent with our directional hypothesis, see *Statistical Comparison of Task vs. Baseline DAs* in Methods Section, Offline single-run analysis for BCI calibration). In between-group analyses and correlations, each participant constitutes one independent observation. In linear mixed-effects model (LMM) analyses, repeated measures of DA across paradigms are treated as within-subject replicates (statistically modelled via random intercepts).

Bivariate associations were evaluated using Pearson’s correlation. Partial correlations were computed to control for group membership, which was entered as a categorical variable using dummy coding. To examine DA across task paradigms within each group, an LMM was fitted with *task paradigm* as a fixed effect and participant as a random intercept to account for repeated measures. Model assumptions of normality, homoscedasticity, and linearity were assessed using residuals versus fitted values and Q–Q plots. Where significant effects of paradigm were identified, estimated marginal means were computed, and pairwise comparisons between paradigms were performed using Holm-adjusted *p* values to control for Type I error. Bivariate associations were evaluated using Pearson’s correlation. Partial correlations were computed to control for group membership, which was entered as a categorical variable using dummy coding.

To predict diagnostic category (UWS, MCS, LIS), a multinomial logistic regression was performed using DA, CRS-R, and WHIM scores as predictors, with injury type (Traumatic = 1; Non-Traumatic = 0) and time since injury (in months) included as covariates. Model performance was evaluated using leave-one-subject-out (LOSO) cross-validation, in which the model was trained on all participants except one, and then used to predict the diagnosis of the held-out participant. This procedure was repeated for each participant to provide subject-level out-of-sample predictions. Overall, accuracy, balanced accuracy, and macro-F1 scores were calculated from the aggregated LOSO predictions, with uncertainty quantified via exact and bootstrap CIs. To explore whether integrating DA and behavioural measures improves diagnostic prediction, we performed a post-hoc ensemble analysis that combined three single-measure models—DA, CRS-R, and WHIM, each fitted with the same covariates as the main multinomial model. Using LOSO, we obtained held-out predictions and class probabilities for every participant from each model. For the ensemble, the final predicted class for each participant was determined by majority vote across the three models; in cases of a three-way tie, we applied probability-based tie-breaking by averaging per-class probabilities and choosing the maximum. Ensemble performance (accuracy, balanced accuracy, macro-F1, and class-wise recall with CIs) was computed from these LOSO-derived predictions to allow direct comparison with the individual predictor measures and the combined multinomial model.

### Topographical analysis methods

Topographical contributions of cortical regions were assessed using data from runs with statistically significant DA. For each task period, CSP weights were multiplied by their corresponding mutual information (MuI) scores to determine the contribution of each electrode. Following the interpretive framework proposed by Haufe et al.^74^, these MuI-weighted CSP values are treated as activation patterns (i.e. estimates of each sensor’s relative contribution to classification performance), rather than as direct representations of cortical source activity. To aid qualitative visualisation, these sensor-level activation patterns were optionally projected into source space using sLORETA; this step was exploratory and not intended as a quantitative inverse solution. Accordingly, scalp-level MuI-weighted CSP maps remain the primary analytical result. Electrodes were grouped by cortical region: frontal (F3, Fz, F4), left-temporal (T7, C3), right-temporal (C4, T8), sensorimotor (C3, Cz, C4), parietal (P3, Pz, P4), and occipital (PO7, Oz, PO8)^75^.

For each paradigm (assessment, training, feedback, Q&A), MuI-weighted CSP values are averaged across electrodes within each cortical region and then across participants, such that each participant contributes one independent observation per region to the group-level comparisons.

### Functional connectivity analysis methods

FC differences between diagnostic groups were evaluated during motor imagery (MI) task performance, using source-level, frequency-specific connectivity measures. The analysis aimed to identify how task-evoked network dynamics varied across diagnostic categories.

The networks of interest included the DMN, FPN, Auditory Network, and Thalamocortical Network, due to their respective roles in endogenous awareness^39,76^, executive control^39,77^, exogenous awareness and auditory processing^33,39,78^, and thalamocortical information integration^39,79,80^. Nodes used for each network were defined as follows—DMN: mPFC, PCC; FPN: left and right dorsolateral prefrontal cortex (l-dlPFC, r-dlPFC), and left and right inferior parietal lobule (l-IPL, r-IPL); Thalamocortical: left and right thalamus (l-Thal, r-Thal); and Auditory: left and right superior temporal gyrus (l-STG, r-STG). The Montreal Neurological Institute (MNI) coordinates for the locations of the nodes are presented in Supporting Information: Additional Exploratory Results, Supplementary Table 6, replicated from the Aubinet et al. study examining resting-state FC in a cohort of MCS patients^39^.

Connectivity was computed within six frequency bands: delta (0.5–4 Hz), theta (4–8 Hz), alpha (8–12 Hz), low-beta (12–18 Hz), high-beta (18–28 Hz), and low-gamma (28–40 Hz). Data were epoched into 1-s windows centred on the peak DA time point within each significant MI trial. Only runs that showed statistically significant DA were included.

EEG source localisation was performed using the New York head model^81^, which incorporates standard electrode positions and a forward model aligned to the MNI template^82^. In this template, the forward model is constrained to a priori selected dipoles with perpendicular orientation to the cortical surface. The dipole locations are represented by the network nodes of interest listed in Supplementary Table 6. To solve the source localisation problem, eLORETA was applied, as it provides zero localisation error under ideal conditions^83,84^ and an estimated localisation error of ~1 cm for general scenarios^85–87^. The inverse solution equations followed from the formulation described in equations Eq. 32 and Eq. 37 by Pascual-Marqui et al.^88^, using a regularisation parameter *α* = 0.01.

FC was estimated between the predefined cortical nodes using imaginary coherence (iCOH). iCOH quantifies the strength of phase coupling between two source signals, while minimising spurious effects from volume conduction and common source artifacts^89^.

The iCOH measure between signals *x*(*t*) and *y*(t) at frequency *f* was computed as:

9 $${{{\rm{iCOH}}}}_{{xy}}(f)=\frac{{{\rm{Im}}}[{S}_{{xy}}(f)]}{\sqrt{{S}_{{xx}}(f)\,{S}_{{yy}}(f)}}$$

where, *S*_*xy*_(*f*) is the cross-spectrum between *x* and *y*, *S*_*xx*_(*f*) and *S*_*yy*_(*f*) are their respective power spectrum values at *f*, and Im[⋅] denotes the imaginary component.

Group-level FC differences were assessed using Spectral Functional Connectivity Difference (SFCD) matrices, derived from the iCOH signed values computed between all 66 paired interactions of twelve predefined cortical nodes (regions) across 6 frequency bands. Firstly, epoched regional activity was concatenated for all the participants in each diagnostic group, then the FC metric was estimated for each group, and SFCD matrices were obtained from the group differences. In parallel, a non-parametric max-statistics approach was implemented using a Monte Carlo permutation test (1000 replications). Here, the condition labels were shuffled prior to the calculation of a surrogate SFCD, finally resulting in a single value corresponding to the maximum absolute difference value of SFCD elements, for each replication. Under the null hypothesis of non-differences between the conditions, this non-parametric max-statistic test provides a distribution for the evaluation of significant differences between the conditions while controlling for multiple comparisons. Significant differences were tested with a threshold of *α* = 0.01.

### Manuscript editing

To improve clarity and conciseness, manuscript editing was aided by ChatGPT (OpenAI, San Francisco, CA). All content was reviewed and verified by the authors for accuracy and appropriateness.

## Results

### Decoding accuracy examples and metrics

AB participants served as a benchmark representing optimal MI-BCI performance, providing a reference framework for interpreting diagnostic group outcomes. Following Phase I (assessment), 8 of 14 UWS, 13 of 17 MCS, 10 of 11 LIS patients, and both AB participants progressed to Phase II (training and feedback; *N* = 33, 73.8% of patients passed assessment). Having completed Phase II, 6 UWS, 12 MCS, 10 LIS, and both AB participants advanced to Phase III (Q&A; *N* = 30, 90% of patients progressed to Q&A). The number of sessions completed by each patient varied between 4 and 13, however, all patients who progressed to Phase III (Q&Q) completed a minimum of 9 sessions. The two AB participants completed 4 and 5 sessions, respectively—one completed 20 runs (including 4 Q&A), and the other completed 15 runs (including 2 Q&A). DA refers to the percentage of correctly classified imagined movements—and throughout the analyses, ‘significant runs’ refer to MI BCI runs where DA during the task period was significantly higher than during the corresponding baseline (one-tailed *t*-test, *α* = 0.05).

Examples of DA dynamics and associated spectral contributions for individual participants from each diagnostic group are provided in the Supporting Information: Methods (Supplementary Fig. 6). As shown there, significant task-related modulation of brain activity is reflected by DA peaks exceeding baseline and permutation-derived chance levels, with corresponding time-frequency maps highlighting SMR contributions, typically within mu and beta bands. These examples serve to illustrate the physiological basis of DA and validate the feature-selection and classification approach used in the main analyses.

**Fig. 4:**
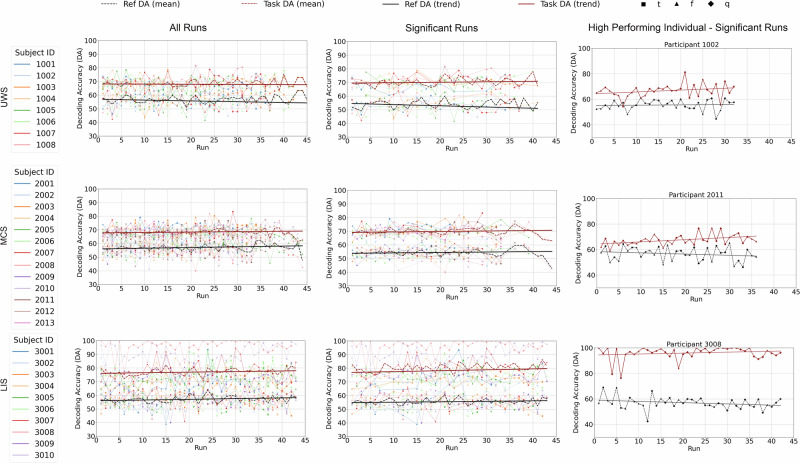
Decoding accuracy (DA) across runs for all participants. Graphs are displayed according to diagnostic category (top row—UWS; middle row—MCS; bottom row—LIS), shown separately for all runs (left-hand column) and runs reaching statistical significance (middle column); group-level trajectories for Ref DA (black) and Task DA (red), including participant traces (see subject ID for each group), mean trajectories (dashed), and participant-averaged linear trends (solid). Representative high-performing individuals, illustrating stable high performance and task-reference separation, are presented in the right-hand column.

Inspection of participant- and run-specific DA, visualised in Fig. 4, reveal substantial variability in both initial performance and longitudinal patterns across runs and sessions. Some LIS participants demonstrated consistently high task DA throughout the protocol, whereas others exhibited gradual improvements over time (see trendlines in Fig. 4). Strong task-related DA was not uniformly accompanied by corresponding changes in reference/baseline DA, particularly for PDoC participants, indicating that task-specific modulation and baseline performance can vary independently across these individuals. Together, these patterns illustrate overall heterogeneity in MI-BCI performance expression across participants, regardless of diagnostic category.

### Group analysis

**Fig. 5:**
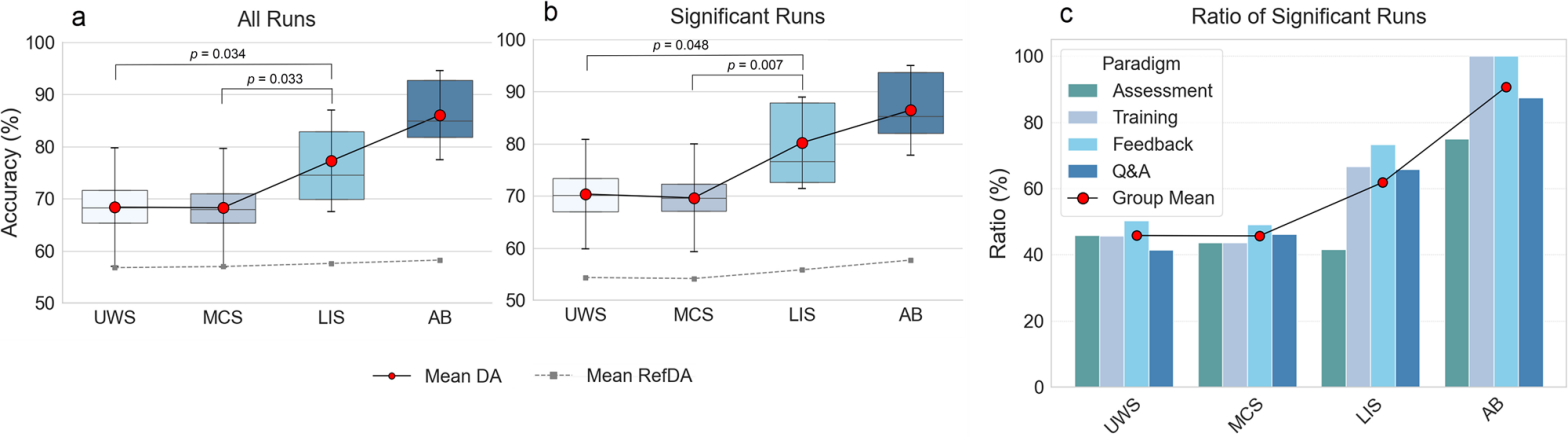
Illustration of the comparison of DA values obtained for each group (UWS, MCS, LIS, AB). DA values obtained from **a** all runs (*n* = 1233 runs/33 participants; UWS = 293/8, MCS = 472/13, LIS = 433/10, AB = 35/2) and **b** runs showing a significant task-related peak (one-tailed *t*-test comparing task vs. reference interval, *α* = 0.05; *n* = 681 runs/31 participants; UWS = 138/8, MCS = 211/13, LIS = 299/10, AB = 33/2). Boxplots display the data distribution for each group, showing the interquartile range (25th–75th percentile) with the median as a horizontal line. Individual data points represent DA values from multiple runs per participant, illustrating within-group variability. Red circles mark group mean DA, with vertical error bars showing ±1 SD. Black circles and solid lines connect mean task-related DA across groups, while grey squares and dashed lines indicate mean DA during the reference interval. **c** The proportion of significant runs per paradigm (assessment, training, feedback, and Q&A), by group. Bars represent the mean percentage of significant runs for each group and paradigm. Individual data points represent proportions from each participant’s runs, illustrating within-group variability. Red points and connecting lines indicate group-level averages across paradigms. Exact *P* values for diagnostic group comparisons are displayed in the figure.

DA was calculated for each participant and paradigm (assessment, training, feedback, Q&A) using the procedure described in the *Offline Single-Run Analysis* section. Subsequent group comparisons were based on these calculated DA values and conducted in two ways: (1) using all runs (Fig. 5a), and (2) using only runs where task-related DA significantly exceeded baseline DA (one-tailed *t*-test, *α* = 0.05; Fig. 5b).

A Kruskal–Wallis test on data from all runs revealed a significant group effect (*H*_(2)_ = 12.047, *p* = 0.0012, *ε*^2^ =  0.36). Dunn post-hoc comparisons with Holm correction showed the LIS group achieved significantly higher DA than both MCS (*p* = 0.033) and UWS (*p* = 0.034) groups, with no significant difference between MCS and UWS. Restricting the analysis to significant runs (Fig. 5b) confirmed group differences (Kruskal–Wallis: (*H*_(2)_ = 10.026, *p* = 0.007, *ε*^2^ = 0.29)). Dunn post hoc tests with Holm correction again showed higher DA in the LIS group compared to MCS (*p* = 0.007), and UWS (*p* = 0.048). Figure 5c shows the proportion of significant runs per paradigm and group. A Kruskal–Wallis test was used to evaluate diagnostic group variance in the proportion of significant runs across paradigms and did not find significant group differences (*H*_(2)_ = 4.65, *p* = 0.098, *ε*^2^ = 0.12).

Descriptive statistics for both tests indicated that the AB group’s mean and median were higher than those of the diagnostic groups, providing a benchmark for interpreting patients’ MI results (Supporting Information, Supplementary Table 1a).

### Q&A question category analysis, across diagnostic groups

In the Q&A phase, the MI-BCI was used primarily as a cognitive assessment tool to examine whether participants with PDoC could intentionally modulate neural activity in response to different questions (in categories including biographical, logical, numerical, and situational questions). While the diagnostic potential of the framework is the focus of the earlier phases, this phase was designed to probe domain-specific awareness and intentional modulation, with the results also providing initial indications of future potential as a communication aid for patients with PDoC and CLIS.

Welch ANOVAs revealed significant group differences (all runs; *F*_(2, 13.69)_ = 4.64, *p* = 0.029, *ω*^2^_adj_ = 0.3), significant runs; (*F*_(2, 13.52)_ = 7.94, *p* = 0.005, *ω*^2^_adj_ = 0.46). Games-Howell post-hoc tests revealed that, with all runs included, LIS had higher DA than MCS (*p* = 0.028) and UWS (*p* = 0.042). When analyses included significant runs only, DA was higher for the UWS group compared to the MCS group (*p* = 0.049) and not compared with the LIS group. A significant difference was also observed between the LIS and MCS groups (*p* = 0.008).

**Fig. 6:**
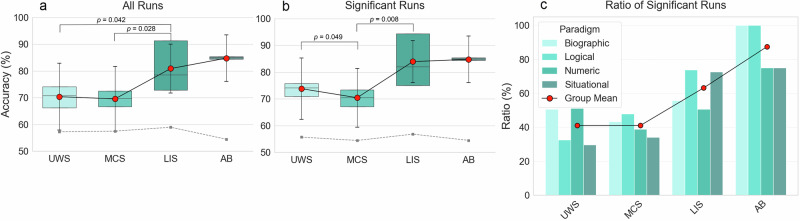
Comparison of decoding accuracy (DA) values obtained for Q&A categories, across groups (UWS, MCS, LIS, AB). DA values obtained from **a** all runs (*n* = 297 runs/30 participants; UWS = 57/6, MCS = 112/12, LIS = 124/10, AB = 4/2) and **b** runs showing a significant task-related peak (one-tailed *t*-test comparing task vs. reference interval, *α* = 0.05; *n* = 169 runs/29 participants; UWS = 26/5, MCS = 51/12, LIS = 88/10, AB = 4/2). Boxplots display the data distribution for each group, showing the interquartile range (25th–75th percentile) with the median as a horizontal line. Individual data points represent DA values from multiple runs per participant, illustrating within-group variability. Red circles mark group mean DA, with vertical error bars showing ±1 SD. Black circles and solid lines connect mean Q&A task-related DA across groups, while grey squares and dashed lines indicate mean DA during the reference interval. **c** The proportion of significant runs per Q&A category (Biographical, Basic Logic (Logic), Numbers and Letters (Numeric), and Situational), by group. Bars represent the mean percentage of significant runs for each group and question category. Individual data points represent proportions from each participant’s runs, illustrating within-group variability. Red points and connecting lines indicate group-level averages across paradigms. Exact *P* values for diagnostic group comparisons are displayed in the figure.

The proportion of significant runs by question category is illustrated in (Fig. 6c). Again, a Kruskal–Wallis test evaluating diagnostic group variance in the proportion of significant runs across question categories did not find significant group differences (*H*_(2)_ = 4.98, *p* = 0.083, *ε*^2^ = 0.12). Descriptive statistics for Q&A session performance, based on both all runs and significant runs only, show higher mean and median values for the AB group, again providing a normative reference for evaluating patient performance (Supporting Information, Supplementary Table 1b). Exploratory analyses of question-level accuracies computed using confusion matrices, across cognitive categories, are provided in Supporting Information: Additional Exploratory Results. Supplementary Table 4a, b summarise the accuracy of decode responses, with interpretive guidance provided in Supplementary Note 3. Supplementary Table 5, together with Supplementary Notes 4 and 5, summarises question-level accuracy rankings by group. UWS and MCS participants achieved mean accuracies of 76–78%, and LIS participants exceeded 80%, whereas accuracies derived from baseline intervals were substantially lower (54–65.5%).

### Paradigm-specific differences in MI-BCI decoding accuracy across diagnostic groups

An LMM was fitted with task paradigm as a fixed effect and participant as a random intercept to test whether MI-BCI performance varied across task paradigms. The model included significant MI-BCI runs only. The assessment paradigm was excluded because it employed a block design, which introduces temporal correlations that may inflate classification performance by capturing slow signal drift rather than stimulus-specific neural responses^90^. As this repeated-measures analysis focuses on within-subject variation, including a structurally different paradigm would confound the comparison. Consequently, the training paradigm (no feedback) was used as the reference condition.

**Fig. 7:**
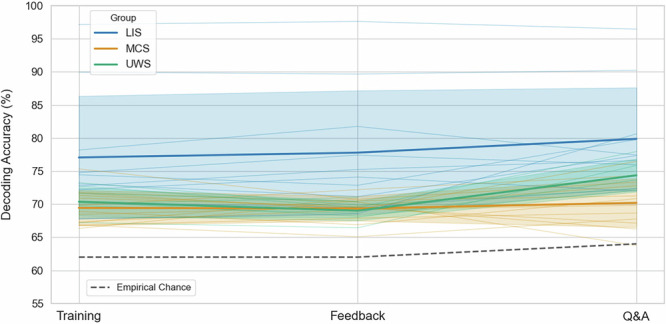
Mean DA across task paradigms (Training, Feedback, and Q&A) for significant runs only, shown separately for diagnostic groups (UWS, *n* = 8; MCS, *n* = 13; LIS, *n* = 10). Thin lines represent individual participants, illustrating within-group variability. Bold lines and shaded bands denote group-level means ±1 SD. The dashed line indicates empirical chance levels for each paradigm, computed using the ‘better-than-random’ binomial rule^66^. Group colours: UWS = green, MCS = orange, LIS = blue.

For the UWS group, adding random intercepts did not significantly improve model fit (*p* = 0.13), whereas including task paradigm as a fixed effect significantly improved fit compared to the null model (*p* = 1.76 × 10^−6^, see green band in Fig. 7). Pairwise comparisons showed higher DA during Q&A compared to training (*p* = 0.002) and feedback (*p* = 1.22 × 10^−6^), with no difference between training and feedback (*p* = 0.12). The Intraclass Correlation Coefficient (ICC) indicated a low between-participant variance (ICC = 0.076).

In contrast, random intercepts significantly improved model fit for both the MCS (*p* = 0.009) and the LIS (*p* = 2.76 × 10^−77^) group, while paradigm had no significant effect on DA in either group (*α* = 0.05). Pairwise comparisons were not significant (*α* = 0.05). However, between-participant variance was low in the MCS group (ICC = 0.103), suggesting that most variability occurred within individuals across runs (as opposed to paradigms); while in the LIS group, ICC was high (0.731), indicating that inter-individual differences were the dominant source of variation in DA.

Between-participant variance was low for the MCS group (ICC = 0.103) but high for the LIS group (ICC = 0.731), indicating that variability in DA for LIS was driven mainly by inter-individual differences. In contrast, MCS variability arose primarily within participants across trials rather than across paradigms.

These results suggest that only the UWS group was sensitive to paradigm differences, whereas inter-individual variability among LIS patients accounted for the majority of variance in DA (see blue band in Fig. 7).

### Analysis of decoding accuracy and CRS-R and WHIM assessment scores

CRS-R and WHIM scores were strongly correlated (Pearson’s *r*(28) = 0.66, *p* = 6.94 × 10^−5^, 95% CI [0.40, 0.82]), indicating substantial agreement between both behavioural assessment measures. Partial correlations between participants’ peak DA and CRS-R and WHIM scores, controlling for diagnostic group, revealed DA was significantly associated with WHIM (partial Pearson’s *r*(28) = 0.43, *p* = 0.02), but not with CRS-R (*p* = 0.89) measures. Group differences in both CRS-R and WHIM scores were significant, indicating both measures significantly differentiated between the diagnostic groups. For CRS-R: Welch ANOVA; *F*_(2, 17.55)_ = 42.61, *p* = 1.84 × 10^−7^, *ω*^2^_adj_ = 0.8 (Games-Howell comparisons; UWS MCS *p* = 0.002; UWS vs LIS *p* = 5.6 × 10^−7^; MCS vs LIS *p* = 0.012). For WHIM: Welch ANOVA; *F*_(2, 15.55)_ = 11.42, *p* = 8.87 × 10^−4^, *ω*^2^_adj_ = 0.53 (Games-Howell comparisons; UWS vs MCS *p* = 0.012; UWS vs LIS *p* = 0.004; MCS vs LIS *p* = 0.018). However, given that the CRS-R and/or WHIM (and/or similar) measures were employed to form the diagnosis, these results are not surprising. At issue is whether diagnoses formed using assessment measures that require overt behavioural responses are accurate.

**Fig. 8:**
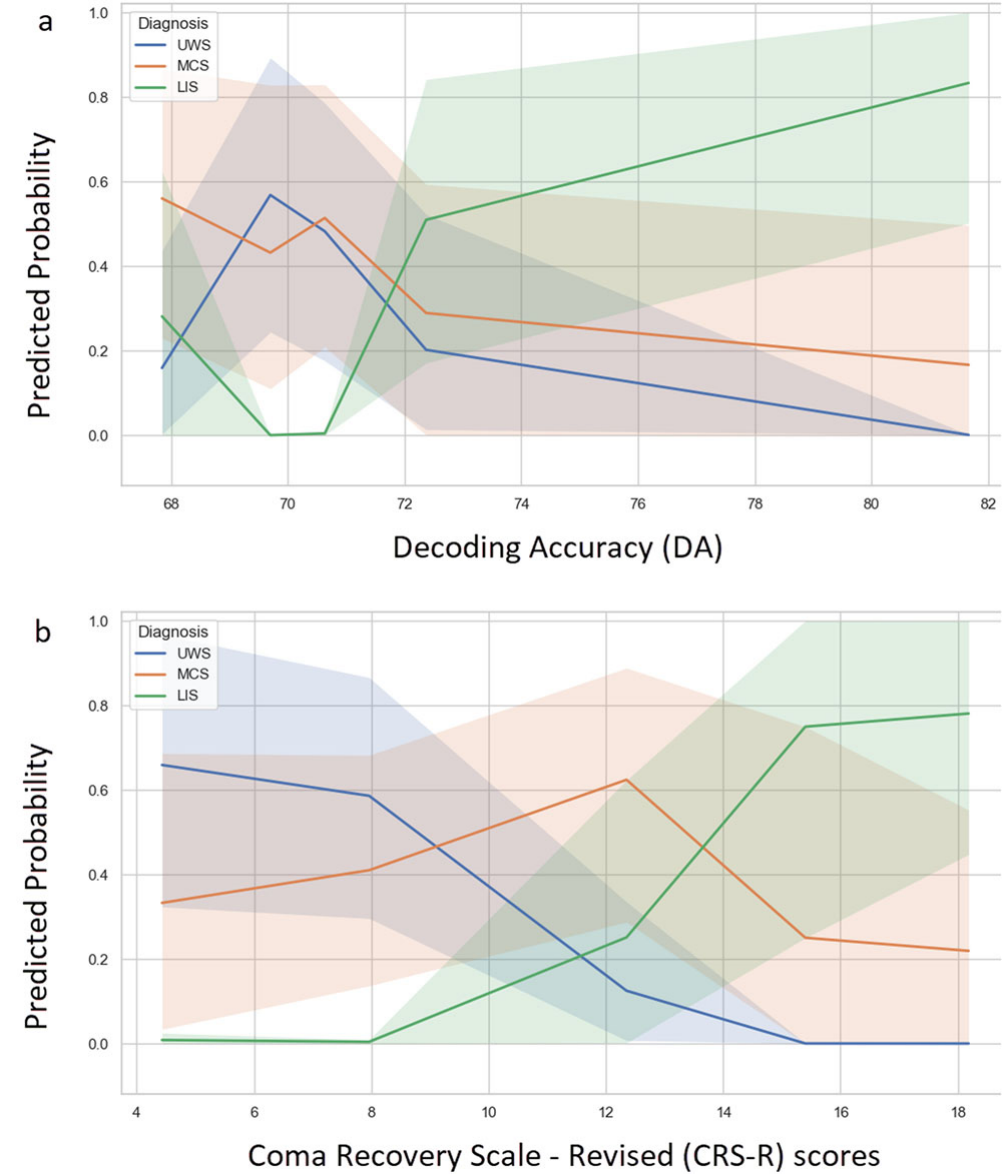
Predicted probabilities of clinical diagnosis (UWS, MCS, LIS) derived from a multinomial logistic regression model as a function of **a** average decoding accuracy (DA) and **b** CRS-R score. The model was fitted using participant-level averages within each diagnostic group. Lines show model-estimated probabilities with 95% bootstrapped confidence intervals. Colours denote diagnostic groups (UWS = blue, MCS = red, LIS = green).

Therefore, to evaluate the predictive value of DA, CRS-R, and WHIM for diagnostic classification, we fitted a multinomial logistic regression model with diagnosis (UWS, MCS, LIS) as the outcome. UWS was set as the reference category. The model included DA, CRS-R, and WHIM as predictors, with injury type (TBI vs. non-TBI) and time since injury (IOT) included as covariates. To provide subject-level external validation, predictive performance was estimated using LOSO cross-validation. This approach ensured that each participant’s diagnosis was predicted from a model trained on all other participants, avoiding within-sample bias. A balanced accuracy of 55.4% (CI: 38–73%) was observed, indicating moderate classification performance across groups, correctly identifying 4/8 UWS, 5/13 MCS, and 7/9 LIS cases. The model performed best for LIS classification (recall = 77.78%, CI: 45–94%), and was weaker for MCS (recall = 38.46%, CI: 18–64%). While predictive metrics were modest and uncertainty wide, the LIS group shows the clearest separation from UWS (see Fig. 8). A complimentary full-sample association analysis found MI-BCI DA has the strongest association with LIS relative to UWS, followed by CRS-R. However, as association analyses are not cross-validated, this finding cannot be taken as evidence of out-of-sample predictive performance.

As an exploratory post hoc analysis, we examined whether combining all three single-measure models, DA, CRS-R, and WHIM (each fitted with the same covariates as the main multinomial model), improves classification relative to any single predictor measure. Using LOSO held-out predictions, single-modality balanced accuracies were 57.3% for DA, 49.4% for CRS-R, and 59.4% for WHIM. A three-model majority-vote ensemble with probability-based tie-breaking achieved a balanced accuracy of 61.81% (95% CI: 43–80.4), macro-F1 = 0.633, and class-wise recalls of 50.0% (UWS), 69.2% (MCS), and 66.7% (LIS).

A ~5% improvement in diagnostic accuracy is achieved when integrating MI-BCI DA as a metric alongside behavioural (CRS-R, WHIM) predictor measures; most notably improving recall for the intermediate MCS group, while acknowledging the wide uncertainty around the estimates. However, the reduced predictive performance for LIS in the integrated ensemble model suggests that MI-BCI DA alone remains the most robust predictor of LIS.

### Brain topography analysis

Topographical maps were visually inspected to identify brain regions contributing most to MI task classification. These maps represent sensor-level activation patterns derived from MuI-weighted CSP values, indicating relative discriminative contributions of each electrode rather than direct neural source estimates^74^. sLORETA projections provide a qualitative visualisation to assess whether scalp-level contributions corresponded to expected sensorimotor regions. Task-related activity was calculated as the difference between common spatial patterns-mutual information (CSP-MuI) weights during task and baseline periods, for each group. In the AB (benchmark) group, activity was consistently localised to sensorimotor and somatosensory regions across paradigms (see Supplementary Fig. 1d). In contrast, the PDoC and LIS groups (particularly PDoC) showed more spatially diverse and less stereotyped patterns (Supplementary Fig. 1a–c).

Group differences in regional CSP-MI activity were evaluated through an analysis of task-related CSP-MI weights across six cortical regions: frontal, left temporal, right temporal, sensorimotor, parietal, and occipital, within each paradigm (full details in Methods; significant results in Supporting Information, Supplementary Data 1F and descriptive statistics in Supplementary Data 1G)

**Fig. 9:**
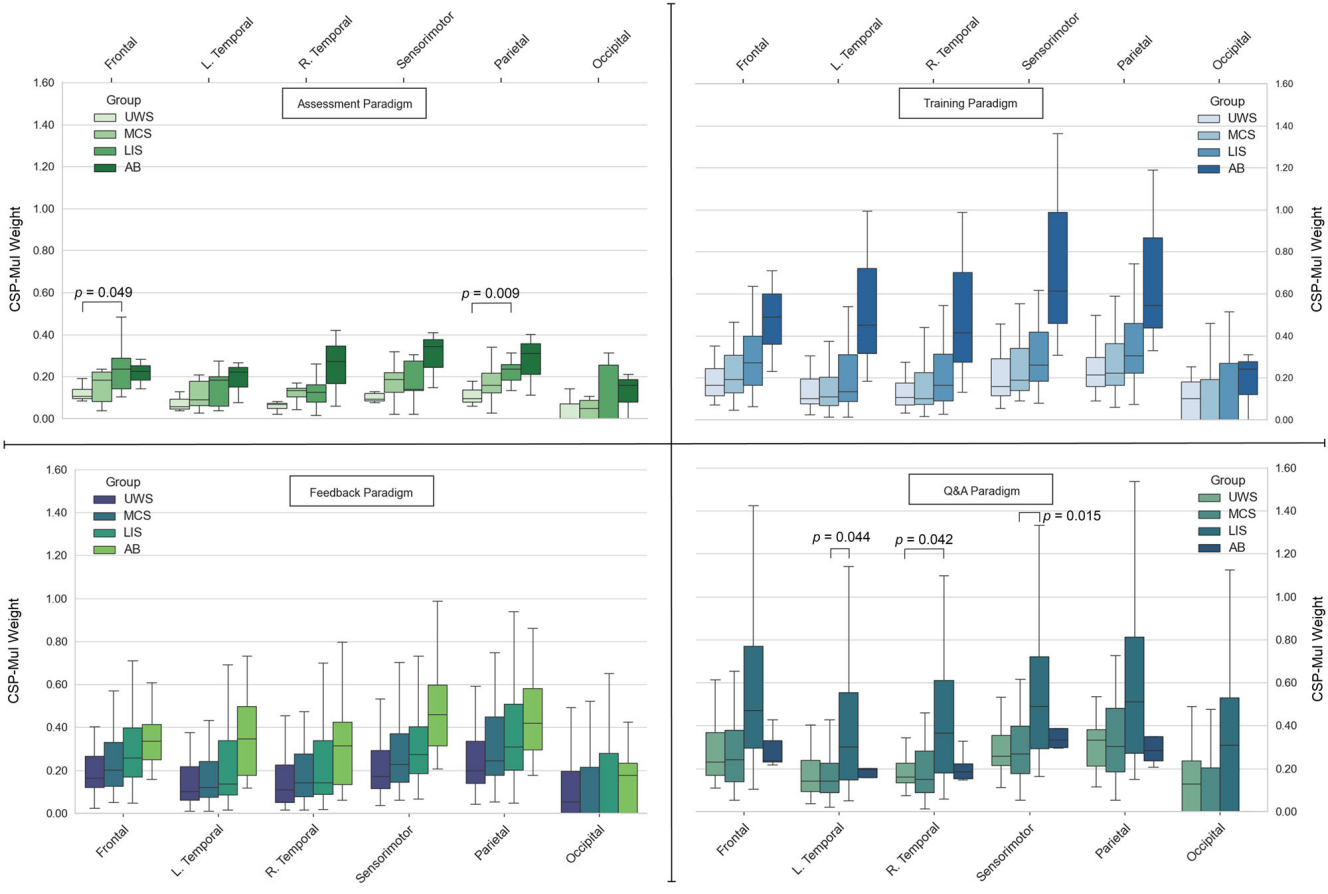
Clustered boxplots illustrating CSP-MuI weights across brain regions for each diagnostic group (UWS, MCS, LIS, AB) and paradigm: Assessment (top left), Training (top right), Feedback (bottom left), and Q&A (bottom right). Each box represents the distribution of CSP-MuI weights across individual runs (multiple runs per participant) within each group and brain region, with boxes showing the interquartile range (25th–75th percentile) and horizontal lines marking the median. Whiskers extend to 1.5× the interquartile range, and outliers are not displayed. Colours denote diagnostic groups as indicated in the legend. Exact P values for diagnostic group comparisons are displayed in the figure. See Supplementary Data 1H for full details on the graphed units of observation.

The results show group differences in MuI-weighted CSP values emerged only in brain regions contributing to MI task classification during the Assessment and Q&A paradigms, with higher MuI-weighted CSP values distinguishing LIS from PDoC groups (as illustrated in Fig. 9). Notably, during the Assessment paradigm the difference in MI-weighted CSP values between LIS and UWS in the parietal region was significant (Welch ANOVA and Games-Howell post-hoc, *F*_(2, 11.8)_ = 7.16, *p* = 0.009, *ω*^2^_adj_ = 0.45, LIS > UWS, *p* = 0.009)—and during the Q&A paradigm, the difference between LIS and MCS in the sensorimotor region was significant (Kruskal–Wallis and Dunn post-hoc tests with Holm correction; *H*_(2)_ = 8.801, *p* = 0.012, *ε*^2^ =  0.28, LIS > MCS, *p* = 0.015). These results suggest that LIS participants produce stronger and more discriminable motor imagery-related activation patterns compared with PDoC patients, particularly early on in the protocol, as well as in the Q&A task, which requires a yes/no response to a question as opposed to simple command following.

### Functional connectivity

Exploratory source-level FC analyses suggested group differences largely consistent with known thalamo-frontal and DMN–FPN disruptions in UWS compared with MCS and LIS. These patterns were most evident in the alpha band, extending into the theta band for UWS-LIS comparisons during the Q&A task (see Supplementary Fig. 7). Given the low-density (16-channel) montage, these source-space results are presented as exploratory only; full details are reported in Supporting Information: Additional Exploratory Results, comprising Supplementary Tables 6 and 7, Supplementary Note 6, and Supplementary Fig. 7. The main conclusions are instead supported by sensor-level and aggregate source-space summaries.

## Discussion

The current study evaluated a multi-phase MI-BCI protocol across four groups: patients with PDoC (UWS (*n* = 14) and MCS (*n* = 17), LIS (*n* = 11)) and able-bodied controls (AB, *n* = 2). The AB participants were included as descriptive benchmarks of the upper bound of decoding performance under limited session exposure, rather than as part of the formal inferential tests, since they completed fewer sessions than the patients. As expected, descriptive statistics show they achieved the highest DAs; however, peak performances in some diagnostic groups occasionally approached these benchmark levels^91,92^. All primary statistical analyses and conclusions remain focused on the diagnostic groups (UWS, MCS, LIS).

The three-phase protocol comprised: Phase I, assessment of initial motor imagery (MI) modulation; Phase II, MI training with neurofeedback; and Phase III, binary question–response testing using closed questions from specific categories, designed to evaluate the potential for cognitive profiling and domain-specific awareness with the MI-BCI framework. Overall, 8/11 UWS, 13/17 MCS, and 10/11 LIS patients (73.8% of recruited patients) achieved significant DA during Phase I, with 6/8 UWS, 12/13 MCS and 10/10 LIS (90% of the patients who passed the Assessment phase) completing phase III, suggesting volitional modulation of brain activity was preserved in a high proportion of patients within this study cohort. An evaluation of diagnostic group differences in the proportion of significant runs, across (a) paradigms and (b) question categories, revealed no significant effects, suggesting comparable (a) motor imagery capacity and (b) potential ability to communicate yes-no answers, across diagnostic groups (see Figs. 5c and 6c). The latter interpretation remains theoretical and requires further empirical testing to assess whether patients can reliably use motor imagery to communicate yes-no responses on a single trial basis however the results demonstrate the average overall response to repetitions of questions may enable, albeit slow, communication.

As illustrated in Fig. 4, group averages can obscure inter-individual variability, masking individuals whose MI-BCI performance is substantially better than is suggested by their diagnostic group mean. The marked heterogeneity in MI-BCI performance across PDoC groups reinforces the growing consensus that preserved covert awareness may be underestimated in patients with PDoC, when assessment relies on tools that require overt behavioural responses^93–95^. Furthermore, the presence of exceptionally high-performing LIS participants highlights the potential for some individuals with LIS to approach maximal DA in a short time-frame. Such cases motivate the development of responder-focused, individually tailored MI-BCI protocols.

In binary motor imagery BCIs, classification accuracies of approximately 70% are commonly adopted as a criterion for reliable control, reflecting statistically significant performance above chance and practical usability, while higher accuracies (≥75–80%) are typically required for sustained online or clinical applications^66,96,97^. Here, we report within-run cross-validation accuracies, as the primary focus is group-level comparative analysis. Nevertheless, the individual results shown in Fig. 4 are indicative of performance that exceeds the commonly adopted criterion level for BCI utilisation. It should be noted, however, that inter-run and inter-session variability, which is more pronounced in these participant groups due to factors such as fluctuating awareness, fatigue, and engagement, makes it more difficult to assume reliable cross-run DA.

The multi-phase protocol adopted in this study, incorporating real-time continuous feedback, is intended as a stepping stone toward training participants to produce reliable single-trial responses. The gradual performance improvements observed in a subset of participants (Fig. 4) suggest that feedback-driven learning and sustained engagement can enhance MI control. These findings indicate that performance may further improve with extended exposure, real-time adaptive feedback, and motivational engagement paradigms.

When statistical analyses of group variance in DA only included significant runs, the difference in overall DA between the LIS and UWS groups was significant (*p* = 0.048), whereas their DA during the Q&A sessions did not differ (*p* = 0.13). In contrast, the difference in DA between the LIS and MCS groups remained consistently significant. While there was no overall DA difference between the MCS and UWS groups (*p* = 0.87), a significant difference emerged between these PDoC groups during the Q&A sessions (*p* = 0.049). This pattern suggests that, when analyses were performed using only significant runs, UWS participants performed marginally better than the MCS group during Q&A sessions, thereby narrowing the apparent MI performance gap with the LIS group. Further support for this finding was provided by the significant variation in Q&A MI performance compared to Training and Feedback, in the UWS group (see Fig. 7). Notably, the Q&A task presented pre-recorded questions spoken by a familiar caregiver or relative. The suggestion is that emotionally salient auditory input potentially enhanced cognitive engagement in UWS patients, consistent with prior evidence showing that meaningful stimuli can activate residual cortical networks in UWS despite widespread disconnection^98^. That this paradigm-specific effect emerged only in UWS indicates a potential moderating effect of emotionally salient stimuli on BCI engagement in lower awareness states. Additionally, or alternatively, the UWS participants may collectively exhibit more awareness than previously assumed, which was not evident through standard behavioural diagnostic tests but became observable over time, via movement-independent responses using the MI-BCI.

Grouping questions into distinct cognitive categories enabled assessment of category-specific DA as a means of probing differential conscious awareness, rather than validating the MI-BCI as a communication channel per se. The randomised presentation and balanced phrasing of Yes/No questions minimised the likelihood of auditory or linguistic confounds in significant runs. In addition, all auditory cues were temporally and procedurally separated from the MI task window, identical across conditions, and too brief to account for sustained decoding differences; occasional tactile re-alerting occurred only between runs. These controls make it unlikely that the observed MI-specific effects were driven by auditory or arousal-related factors, although we acknowledge this as a potential limitation. These preliminary findings indicate that MI-BCI responses may offer a window into preserved cognitive processing in participants with PDoC. Future studies will implement counterbalanced Yes/No mappings, auditory-control conditions, and formal multiplicity corrections to further substantiate and refine this approach.

A multinomial modelling framework was used to examine the relative contributions of MI-BCI, DA and behavioural measures to diagnostic classification. Under LOSO cross-validation, overall classification accuracy was modest (Balanced Accuracy; 55.41%), with DA and CRS-R contributing independently to differentiation from UWS. Whereas CRS-R scores showed lower sensitivity for LIS, DA achieved higher recall for LIS cases (77.8%) and provided clearer separation of LIS from UWS and MCS in predicted probabilities compared to CRS-R (see Fig. 8). A post-hoc ensemble combining DA, CRS-R and WHIM predictions improved balanced accuracy to 63.21% (95% CI: 44–80%), with MCS predictive accuracy increasing from 38.46% in the multinomial model to 69.23% in the ensemble. Previous MI-BCI studies in PDoC populations have primarily used the CRS-R as the comparative behavioural measure, as this measure is considered the clinical gold standard for assessing responsiveness^99–101^. However, by including the WHIM, our findings reveal that DA correlates significantly with WHIM scores (*p* = 0.02) but not with CRS-R scores (*p* = 0.89). The WHIM contributed the least to diagnostic classification in the multinomial logistic regression, whereas both DA and CRS-R showed significant predictive power. Nonetheless, combining all three measures modestly improved model performance. Given that the CRS-R aggregates behaviour into broad categorical levels, while the WHIM provides a finer-grained, context-sensitive measure of spontaneous and goal-directed responses, these findings suggest that WHIM may more closely reflect the neural substrates underlying covert command-following detected by MI-BCI. At the same time, each measure appears to capture distinct aspects of residual function, together improving diagnostic sensitivity. Together, these results suggest that MI-BCI can complement existing clinical standards for the assessment of patients with PDoC, improving diagnostic accuracy.

Regarding analyses at the sensor and source space, analysis of spatial features derived from task-related activity over sensorimotor and parietal cortices further supports distinctions between diagnostic groups. CSP-MuI weights applied for feature selection over these motor-associative areas of cortex differentiated LIS and PDoC groups, specifically distinguishing LIS from UWS during the Assessment paradigm (parietal; *p* = 0.009) and LIS from MCS during the Q&A paradigm (sensorimotor; *p* = 0.015). These findings implicate the sensorimotor-parietal network in the preserved capacity for motor imagery and volitional modulation in LIS, and more broadly confirm the role of these networks in MI-BCI control, demonstrating that BCI performance reflects physiologically meaningful activation, rather than random variance. Exploratory FC analyses suggested network-level alterations in UWS relative to MCS and LIS, most prominently involving thalamo-frontal and DMN–FPN interactions (see Supporting Information: Additional Exploratory Results, Supplementary Tables 6 and 7, Supplementary Note 6, and Supplementary Fig. 7). These patterns were most evident during cognitively demanding tasks such as the Q&A paradigm, and align with known mechanisms of impaired awareness in DoC^38,41,42^. Although limited by the 16-channel montage, these exploratory findings support the idea that task-based FC can reveal context-specific neural disruptions relevant to residual awareness, complementing the main sensor-level and behavioural results.

Taken together, the convergence of decoding, spatial, and preliminary FC findings underscores the diagnostic potential of this multi-phase MI-BCI protocol as a clinically viable tool to augment existing behaviour-based protocols. However, several limitations should be acknowledged. While this study represents the most comprehensive MI-BCI protocol trialled in a PDoC population to date, involving fifteen NHS hospitals in the UK and the NRH in Ireland in patient recruitment, unequal group sizes constrained some statistical comparisons, though the analysis of all MI-BCI runs achieved 91% power^102^, and large effect sizes were found for the analyses of group variance based on MI-BCI DA values. Although improvements were made to BCI parameter updating and paradigm sequencing compared to previous work^18^, future performance may benefit from fully adaptive BCI systems capable of real-time adjustment to a participant’s mental state^103^.

EEG instability due to non-stationary brain states, linked to fatigue, arousal, medication, or learning, remains a challenge, often resulting in covariate shift^104,105^. Changes were observed in the evolution of frequency responses and feature importance, sometimes introducing features that degraded performance. DA could likely be improved through more fine-grained, participant-specific band optimisation, with adaptive classifiers^106^, feature adaptation^107^, or data space adaptation^108,109^ also representing promising strategies to mitigate these effects. The current protocol included per-session recalibration; however, if a prior session’s BCI setup outperformed the new calibration, the previous model was reused. While this maximised neurofeedback quality, it may have compromised patient learning, as classifier features could vary across sessions. Future work should develop globally optimised training data selection and automation for classifier updates.

Furthermore, although concurrent EMG was not recorded, visual inspection did not reveal sustained artefacts consistent with muscle activity. Given the severe motor impairment of participants, systematic EMG contamination is unlikely. Nevertheless, the integration of EMG and eye-tracking would benefit future research to further rule out residual movement influences.

Additionally, while the FC analysis revealed interactions involving thalamo-frontal regions, these findings are exploratory and should be interpreted cautiously given the 16-channel EEG configuration and limited spatial resolution. Future work employing high-density EEG or beamformer-based inverse solutions (e.g. Atlantis Source Connectivity Toolbox)^110–112^, will be required to substantiate these observations. Additionally, while imaginary coherence (iCOH) is robust to volume conduction and well-suited for non-parametric testing, it has interpretive limitations. iCOH detects non-zero phase-lagged coupling but cannot reliably infer temporal directionality—signals with vastly different delays (e.g. 10 vs 90 ms) can yield identical values^113^. Although faster communication is often assumed in low-frequency bands, this assumption is not always valid. Techniques like Phase Slope Index or Granger Causality could offer richer insights but are themselves limited (e.g. GC’s vulnerability to volume conduction^114^).

Although anecdotal, feedback from families suggests a possible therapeutic benefit from engaging with the MI-BCI system. Some patients appeared to become aware of their influence on the system, potentially stimulating cognitive activity. This aligns with the observation that FC differences between UWS and MCS patients during the Q&A task were similar to those observed between UWS and LIS differences, suggesting possible adaptive effects that merit further investigation (Supplementary Fig. 7).

Regarding the support for the integration of DA and the CRS-R to improve diagnostic prediction, these findings should be interpreted cautiously, given the broad CIs, and the potential instability of parameter estimates in multinomial models with small group counts. Although LOSO cross-validation mitigates within-sample bias, it remains an internal validation procedure; external replication in larger, independent cohorts will be essential to confirm the robustness and generalisability of these classification patterns.

A key limitation of the study design concerns the inclusion threshold applied during Phase I: participants had to achieve a mean DA > 70% and/or a significant DA peak (one-tailed *t*-test, *α *= 0.05) during the task versus baseline. Given that participants were initially classified using behavioural assessments, known to produce misdiagnoses, especially in UWS^10,11^, this introduces diagnostic circularity. To truly evaluate the MI-BCI’s potential, future protocols should use updated clinical diagnoses, blind researchers to these diagnoses during testing, and remove assessment thresholds to avoid sampling bias. A second related important limitation, which warrants acknowledgement, is that the paired-samples *t-*test used to assess whether DA during the task exceeded baseline DA at the fold level does not assume independence of CV folds. This test was retained for descriptive consistency, and comparison with alternative statistical methods indicated that our conclusions are robust to the choice of inference procedure (see Supporting Information: Methods, Supplementary Table 3).

While the exploratory analysis of the question-level accuracy attained for the illustrative case is of considerable interest in view of the potential for communication-relevant applications, the findings should be interpreted cautiously, as the analysis is based on a snapshot sample of one per group, and the confusion matrices were computed from offline results. Future work will be needed to test the reliability and practicality of MI-BCI use for communication in larger and more systematic designs.

Furthermore, practical constraints also introduced variability. While improving ecological validity, conducting sessions across hospitals, care homes, and private homes also created inconsistencies in intersession intervals and testing environments. The inherent instability of PDoC patients further complicates standardisation. Ideally, recordings would occur during each patient’s considered peak arousal window, but this is often impractical due to logistical constraints. While our protocol already included multiple recording sessions per participant, collecting data across additional sessions could further increase the likelihood of assessing each patient during their optimal state of awareness. Runs identified as significant, or sessions yielding the highest number of significant runs, may indicate periods of peak arousal or cognitive responsiveness, suggesting that denser longitudinal sampling could enhance sensitivity to intra-individual fluctuations in conscious state.

In conclusion, despite some limitations, the study findings highlight the transformative potential of this multi-phase motor imagery BCI protocol in reshaping diagnostic practice for patients with severe motor and cognitive impairments. By uncovering covert cognitive capacities often missed by standard behavioural assessments, the protocol enables a more nuanced differentiation across clinical categories. Therefore, integrating DA as a metric, with CRS-R and WHIM behavioural indices, offers a more comprehensive assessment framework, improving diagnostic precision and sensitivity to residual awareness, particularly for LIS classifications. Furthermore, analysis of MI-weighted CSP values over motor-associative cortical regions indicates that motor imagery-related activation is preserved to a greater extent in LIS than in PDoC, consistent with decoding results showing that LIS participants consistently achieved the highest DA, a generally significant group effect confirming preserved volitional control despite total motor paralysis. In contrast, paradigm-specific neural signatures proved more informative for distinguishing patients with PDoC. Notably, a significant boost in DA among UWS patients during the familiar-voice Q&A paradigm suggests that emotionally salient stimuli can transiently unlock hidden awareness. Although the FC analysis was exploratory, distinct thalamo–frontal and DMN–FPN network alterations were revealed in UWS, and to a lesser extent, in MCS, relative to LIS. These patterns are consistent with established markers of impaired awareness in DoC^38,41,42^. Together, DA, CSP-MuI features, and connectivity metrics capture complementary facets of brain function, indicating that an interactive, personalised BCI approach holds promise as a scalable, movement-independent diagnostic tool—that may eventually support clinical decision-making and give voice to those otherwise unable to communicate.

## Supplementary information

Supporting Information

Description of Additional Supplementary Files

Supplementary Data

## Data Availability

The data that support the findings of this study are available here: 10.15125/BATH-01632^115^. This anonymised dataset includes electroencephalography (EEG) recordings, descriptive variables, and session-level Coma Recovery Scale-Revised (CRS-R) and Wessex Head Injury Matrix (WHIM) scores. All data contained within the repository are provided under controlled access. Access will be granted upon reasonable request, which should include a brief description of the proposed research use, confirmation of relevant ethical approval where applicable, and agreement to data use conditions that prohibit data redistribution or use beyond the approved scope. Requests should be directed to the corresponding author (D.C.) and will be reviewed on a case-by-case basis. A response will typically be provided within 2 weeks of receipt of a complete request. The source data are graphed in Figs. 4–9 and Supplementary Fig. 7 are provided in Supplementary Data 1 (worksheets Supplementary Data A–E, H, I, respectively).

## Code availability

The offline signal processing code used during the current study to analyse a single participant/run is available here: 10.15125/BATH-01632^115^. The code contained in the repository provides an analysis framework developed for the EEG data files, also in this repository. Therefore, this code is provided under controlled access to ensure appropriate use within the approved methodological context. Access will be granted upon reasonable request, which should include a brief description of the proposed research use, confirmation of relevant ethical approval where applicable, and agreement to data use conditions that prohibit data redistribution or use beyond the approved scope. Requests should be directed to the corresponding author (D.C.) and will be reviewed on a case-by-case basis. A response will typically be provided within 2 weeks of receipt of a complete request. Statistical analyses were performed using R-studio (version 4.4.2)^73^.
