# Supplementary material for: Advancing EEG-based assessment of consciousness and cognition in prolonged disorders of consciousness
Source: Commun Med (Lond). 2026 Apr 17;6:344. doi: 10.1038/s43856-026-01574-x (PMC13272656; doi:10.1038/s43856-026-01574-x)
Supplement: Supplementary file 1 — Supporting Information [file 43856_2026_1574_MOESM1_ESM.docx]

**Advancing EEG-Based Assessment of Consciousness and Cognition in Prolonged Disorders of Consciousness**

**Supporting Information**

**Authors:** Naomi du Bois^1,^, Attila Korik^1,^, Stephanie Hodge^2^, Leah Hudson^2^, Ainjila S. Elahi^2^, Alain Bigirimana^3^, Natalie Dayan^2^, Jose M. Sanchez-Bornot^2^, Alison McCann^4^, Kudret Yelden^5^, Lloyd Bradley^6^, Krishnan P. S. Nair ^7^, Simon Judge^8^, Damon Hoad^9^, Emma Vines^9^, Venu Harilal^10^, Sheryl Parke^10^, Paul Johnson^11^, Jacqueline Pogue^12^, Emma Dodds^13^, Abayomi Salawu^14^, Raymond Carson^4^, Karl McCreadie^2^, Jacqueline Stow^4^, Jacinta McElligott^4^, Aine Carroll^4, 15^, and Damien Coyle^1, 2 *^

**Affiliations**

^1^ Bath Institute for the Augmented Human (IAH), University of Bath, Bath, BA2 7AY, UK

^2^ Intelligent Systems Research Centre, Ulster University, BT48 7JL, UK

^3^ Queens University Belfast, Belfast, BT7 1NN, UK

^4^ National Rehabilitation Hospital, Dublin, A96 RPN4, IRE

^5^ Kings College Hospital, London, SE5 9RS, UK

^6^ Royal Hospital for Neuro-disability, Putney, London, SW15 3SW, UK

^7^ Sheffield Teaching Hospitals NHS Foundation Trust, Sheffield, S10 2JF, UK

^8^ Barnsley Hospital NHS Foundation Trust, Barnsley, S75 2EP, UK

^9^ South Warwickshire University NHS Foundation Trust, Rugby, CV21 3SR, UK

^10^ Norfolk Community Health and Care NHS Trust, Norwich, NR2 3TU, UK

^11^ Western Health and Social Care Trust, Derry, BT47 6SB, UK

^12^ Northern Health and Social Care Trust, Antrim, BT41 2RL, UK

^13^ Oxford University Hospitals NHS Foundation Trust, Oxfordshire, OX3 9DU, UK

^14^ Hull University Teaching Hospitals NHS Trust, Hull, HU3 2JZ, UK

^15^ Health Sciences Centre, University College Dublin, Dublin 4, IRE

* Prof Damien Coyle (corresponding author)

**Affiliation:** Director of the Bath Institute for the Augmented Human

**Address:** Bath Institute for the Augmented Human (IAH), University of Bath, Claverton Down, Bath, BA2 7AY, UK

**Email:** [dhc30@bath.ac.uk](mailto:dhc30@bath.ac.uk)

**Phone:** +441225386896

# Supporting Information: Supplementary Tables

**Supplementary Table 1. Descriptive Statistics** **for all groups (UWS, MCS, LIS, AB): (a)** MI-BCI DA for all runs and significant runs. **(b)** MI-BCI DA for all runs and significant runs in the Q&A sessions. Median, mean and standard deviation (SD) for all runs, and significant runs, obtained for all groups (UWS, MCS, LIS, AB).

| (a) | Statistical values based on all runs | | | | Statistical values based on significant runs | | | |
| --- | --- | --- | --- | --- | --- | --- | --- | --- |
|  | n | Mean | Median | *SD* | n | Mean | Median | *SD* |
| AB | 2 | 86.70 | 86.70 | 6.63 | 2 | 86.86 | 86.86 | 6.40 |
| LIS | 10 | 77.04 | 74.81 | 8.97 | 10 | 78.23 | 75.27 | 8.27 |
| MCS | 13 | 68.35 | 68.05 | 0.95 | 13 | 69.67 | 69.84 | 1.76 |
| UWS | 8 | 68.39 | 68.51 | 1.22 | 8 | 70.22 | 70.28 | 1.60 |

| (b) | Statistical values based on all Q&A runs | | | | Statistical values based on significant Q&A runs | | | |
| --- | --- | --- | --- | --- | --- | --- | --- | --- |
|  | n | Mean | Median | *SD* | n | Mean | Median | *SD* |
| AB | 2 | 85.83 | 85.83 | 4.71 | 2 | 87.01 | 87.01 | 3.04 |
| LIS | 10 | 78.35 | 74.93 | 8.77 | 10 | 80.06 | 77.38 | 7.77 |
| MCS | 12 | 69.57 | 69.54 | 1.97 | 12 | 70.20 | 70.33 | 3.68 |
| UWS | 6 | 70.21 | 70.31 | 1.68 | 5 | 74.37 | 73.66 | 2.49 |

Supplementary Table 2. Description of the terms for the confusion matrix used to determine the accuracy of the decoded responses in the Q&A task, per question category and group.

|  | Predicted “Yes” | Predicted “No” |
| --- | --- | --- |
| True “Yes” | True Positive (TP) | False Negative (FN) |
| True “No” | False Positive (FP) | True Negative (TN) |

# Supporting Information: Supplementary Figures


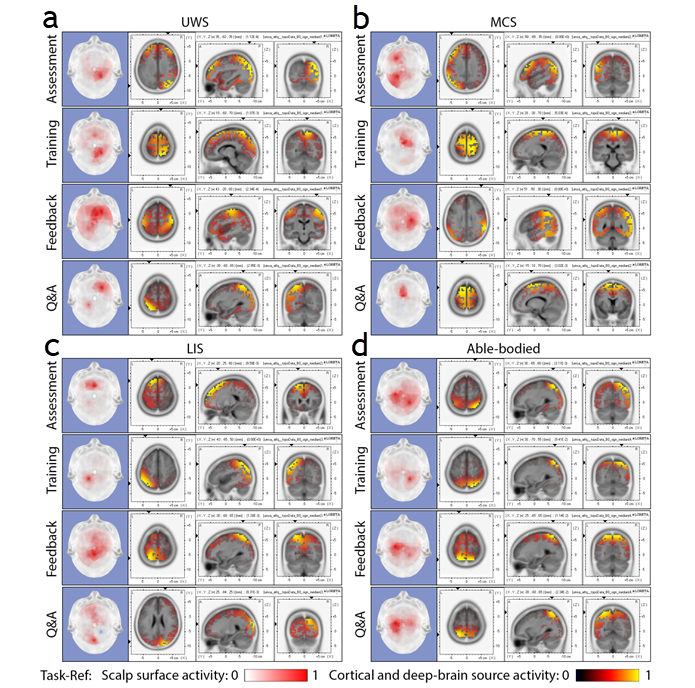


Supplementary Figure 1. Topographical maps of task-related brain activity for each diagnostic group: (a) UWS, (b) MCS, (c) LIS, and (d) AB. Maps reflect differences in CSP–MuI weights between task and baseline periods, averaged across runs showing significant task-related decoding accuracy (DA). Each group’s results are shown across all paradigms (Assessment, Training, Feedback, Q&A). Runs were included based on significantly higher peak DA during task vs. baseline, determined using a one-tailed paired-sample *t*-test (α = 0.05). No additional voxelwise or multiple-comparison correction was applied, as these topographies are descriptive activation patterns (see Methods). Corresponding baseline, task, and difference maps are shown in Supplementary Figures 2-5.


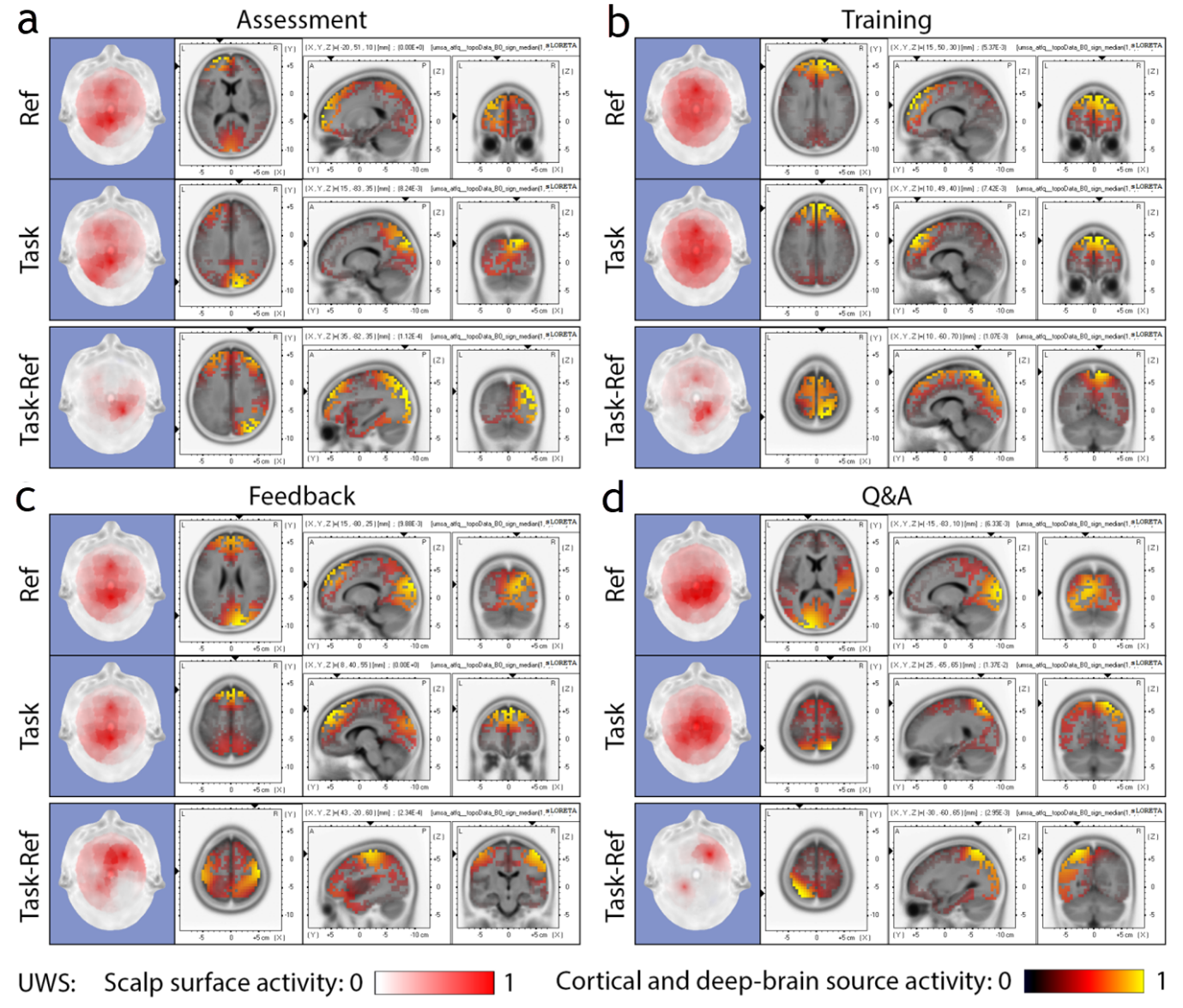


Supplementary Figure 2. Topographical maps from UWS patients: Topographical maps obtained from the four paradigms (assessment, training, feedback, Q&A) are presented in a-d, respectively. In each sub-plot (a-d) results are presented using MuI weighted CSP patterns calculated from the reference baseline period, task period, and as a difference of the task versus reference periods, separately (indicated with text labels). Topographical maps presented in this figure are computed based on runs that provided a significantly higher DA peak during the task period compared to DA peak obtained in the reference baseline period (one-tailed paired-sample *t*-test, α = 0.05). These maps are descriptive visualisations of activation patterns; therefore, no additional statistical tests or corrections for multiple comparisons were applied.


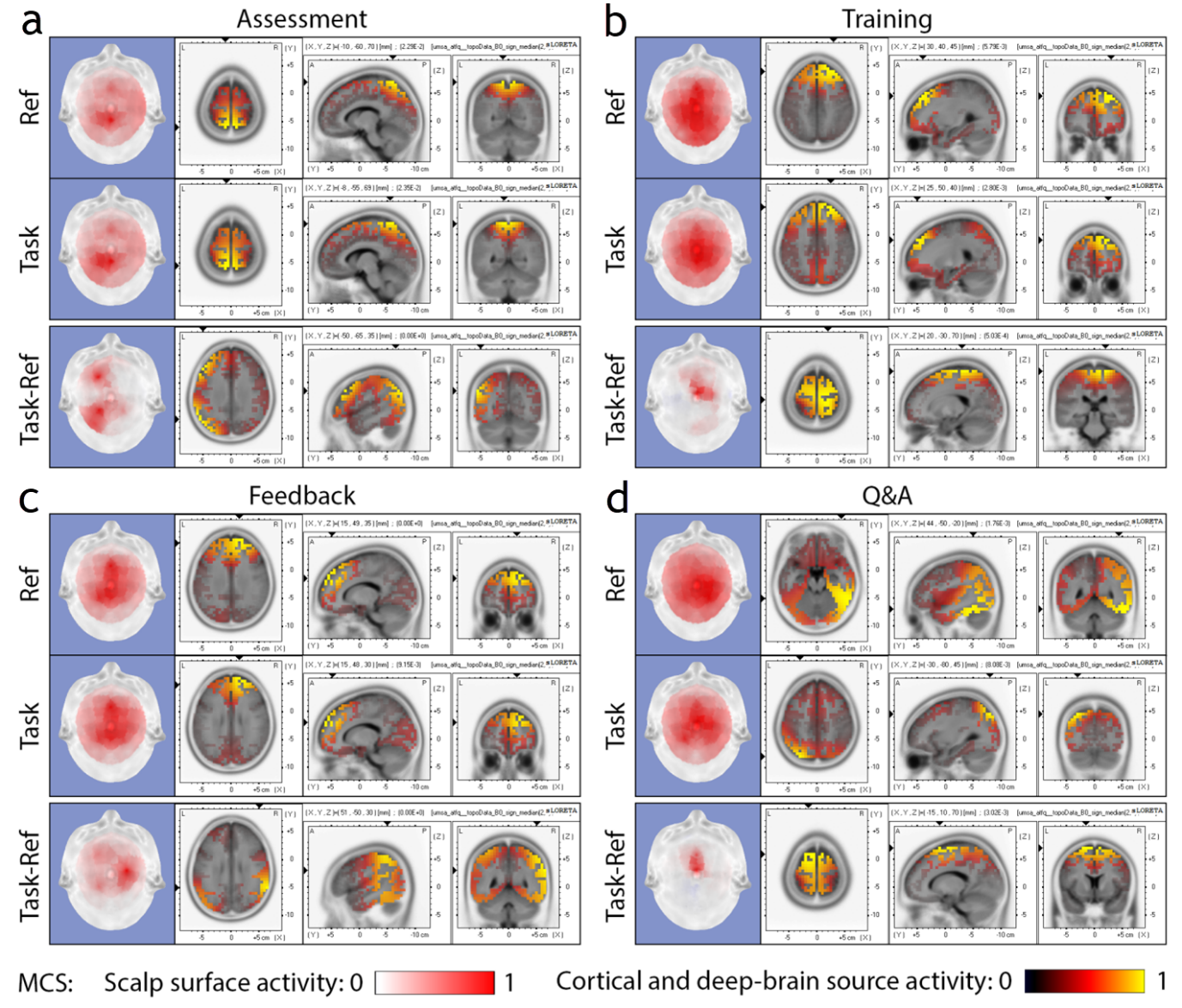


Supplementary Figure 3. Topographical maps from MCS patients. Topographical maps obtained from the four paradigms (assessment, training, feedback, Q&A) are presented in a-d, respectively. In each sub-plot (a-d) results are presented using MuI weighted CSP patterns calculated from the reference baseline period, task period, and as a difference of the task versus reference periods, separately (indicated with text labels). Topographical maps presented in this figure are computed based on runs that provided a significantly higher DA peak during the task period compared to DA peak obtained in the reference baseline period (one-tailed paired-sample *t*-test, α = 0.05). These maps are descriptive visualisations of activation patterns; therefore, no additional statistical tests or corrections for multiple comparisons were applied.


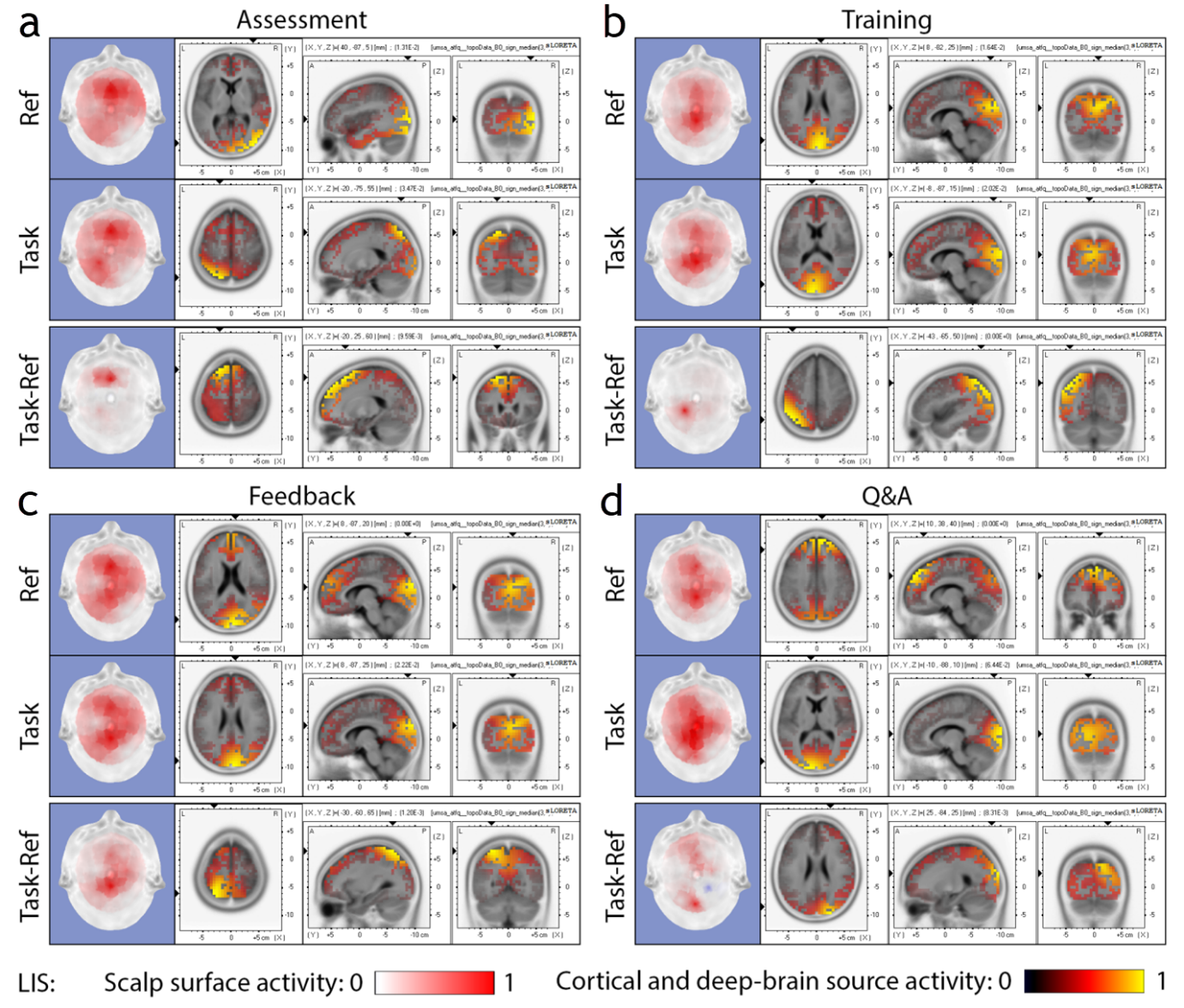


Supplementary Figure 4. Topographical maps from LIS patients: Topographical maps obtained from the four paradigms (assessment, training, feedback, Q&A) are presented in a-d, respectively. In each sub-plot (a-d) results are presented using MuI weighted CSP patterns calculated from the reference baseline period, task period, and as a difference of the task versus reference periods, separately (indicated with text labels). Topographical maps presented in this figure are computed based on runs that provided a significantly higher DA peak during the task period compared to DA peak obtained in the reference baseline period (one-tailed paired-sample *t*-test, α = 0.05). These maps are descriptive visualisations of activation patterns; therefore, no additional statistical tests or corrections for multiple comparisons were applied.


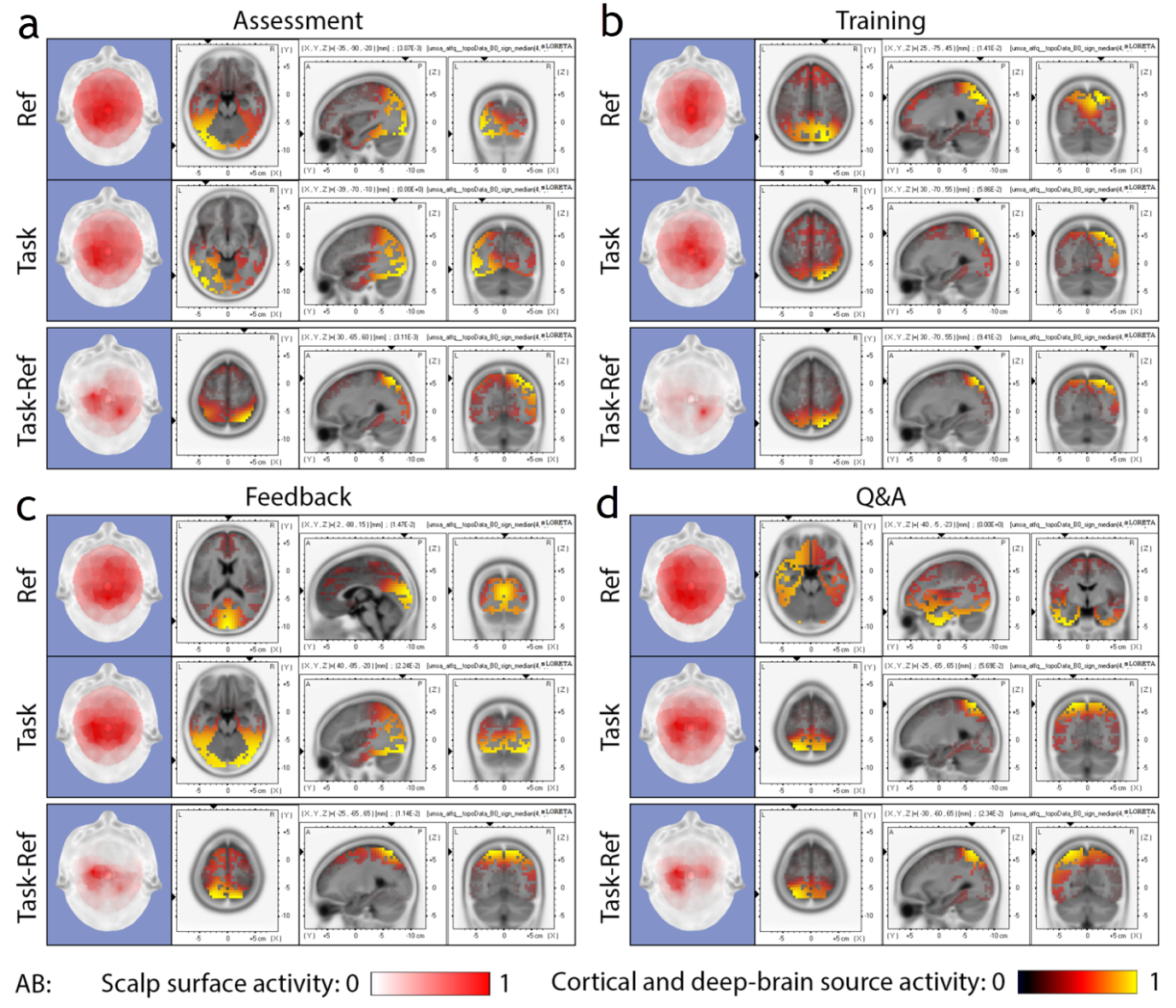


Supplementary Figure 5.Topographical maps from able-bodied (AB) participants: Topographical maps obtained from the four paradigms (assessment, training, feedback, Q&A) are presented in a-d, respectively. In each sub-plot (a-d) results are presented using MuI weighted CSP patterns calculated from the reference baseline period, task period, and as a difference of the task versus reference periods, separately (indicated with text labels). Topographical maps presented in this figure are computed based on runs that provided a significantly higher DA peak during the task period compared to DA peak obtained in the reference baseline period (one-tailed paired-sample *t*-test, α = 0.05). These maps are descriptive visualisations of activation patterns; therefore, no additional statistical tests or corrections for multiple comparisons were applied.

# Supporting Information: Methods

Supplementary Table 3. Contingency table summarising agreement in significance of runs across methods. Each table cross-tabulates the number of runs (*N* = 137, taken from a subset of participants) classified as significant (*p* < 0.05) or not significant (*p* > 0.05) by two different statistical procedures (denoted in merged rows). These counts provide a descriptive comparison of method concordance.

| Paired-samples I-test versus McNemar’s *χ²* test | | |
| --- | --- | --- |
| McNemar’s  *t*-test | *p* < 0.05 | *p* > 0.05 |
| *p* < 0.05 | 73 | 1 |
| *p* > 0.05 | 44 | 19 |
| Paired-samples I-test versus 100 label permutations | | |
| 100 perm.  *t*-test | *p* < 0.05 | *p* > 0.05 |
| *p* < 0.05 | 64 | 10 |
| *p* > 0.05 | 36 | 27 |
| McNemar’s χ² test versus 100 label permutations | | |
| 100 perm.  McNemar’s | *p* < 0.05 | *p* > 0.05 |
| *p* < 0.05 | 93 | 24 |
| *p* > 0.05 | 7 | 13 |


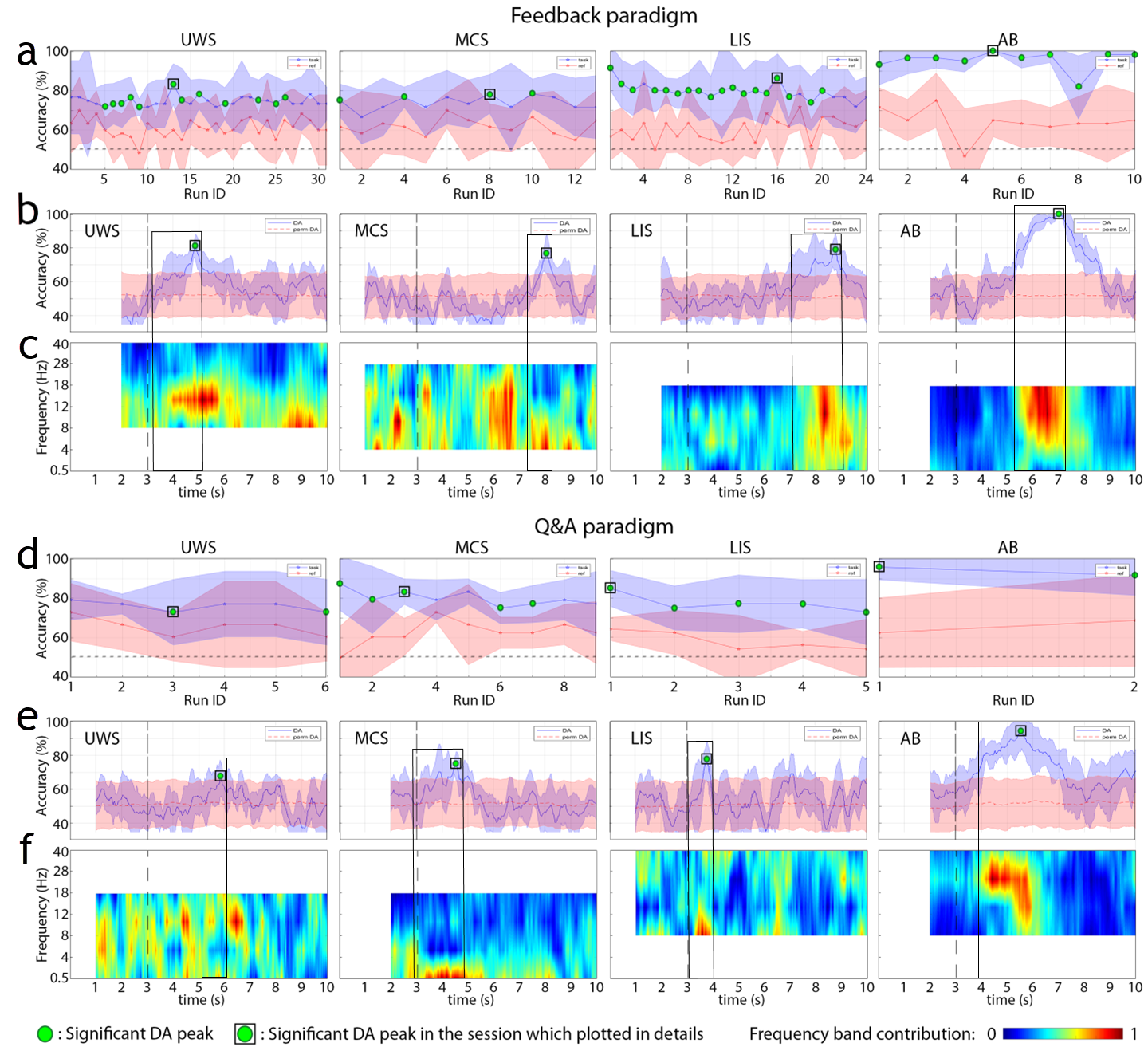


Supplementary Figure 6. Accuracy plots and frequency maps illustrating the results of one participant, from each group (UWS, MCS, LIS, AB). Panels a-c show results from the feedback paradigm; d-f show results from the Q&A paradigm. a and d: Peak decoding accuracy (DA) per run during task (blue) vs. baseline (red) periods. Solid lines represent mean DA; shaded areas indicate standard deviation. Green dots mark significant differences (one-tailed *t*-test comparing task vs. reference interval, α = 0.05). b and e: Time-varying DA curves across a 60-trial run, comparing the original data (blue) with a permutation-based chance distribution (red). Shaded areas show standard deviation; green squares indicate peak DA. c and f: Frequency maps showing band-specific contributions (via CSP-MuI weights) to DA over time. Vertical rectangles in b/e and c/f indicate the 1 s or 2 s sliding window used to derive peak DA, illustrating the time–frequency relationship.

Supplementary Note 1. Paradigm Specific Instructions

## Assessment Paradigm

[Example – “Right” arm versus “Left” arm *Assessment* run]

“We will now begin the session.

You will hear a series of instructions and beeps.

Every instruction will be followed by fifteen beeps.

Every time you hear a beep – imagine the movement described in the instruction.

Concentrate on the way your muscles would feel if you were really performing this movement.

Try to do this as soon as you hear a beep and then relax.

If you do not wish to participate – simply do not imagine anything.

Hear we go.”

[Example – for a “Left” arm imagined movement block]

“Every time you hear a beep imagine lifting a weight with your “Left” arm – try to do this as soon as you hear a beep and then relax.”

Trial begins with a “Beep” – and ends with the instruction to “Relax” (duration between “Beep” and “Relax”, is 10 s).

## Training Paradigm

[Example – “Right” arm versus “Left” arm *Training* run]

“We will now begin a training session.

When you hear the word “Right” try to imagine moving your “Right” arm.

When you hear the word “Left” try to imagine moving your “Left” arm.

Try to be consistent all the time.

Here we go.”

[Example – for a “Right” trial in a “Right” arm versus “Left” arm *Training* run]

Trial begins with the word “Right” – and ends with the instruction to “Relax” (duration between “Right” and “Relax”, is 5 s).

## Feedback Paradigm

[Example – “Right” arm versus “Left” arm *Feedback* run]

“We will now begin a session with sounds.

When you hear the word “Right” try to imagine moving your “Right” arm.

When you hear the word “Left” try to imagine moving your “Left” arm.

Don’t worry if the sound does not move as expected.

Try to be consistent all the time.

Here we go.”

[Example – for a “Right” trial in a “Right” arm versus “Left” arm *Feedback* run]

Trial begins with a “Right” – and ends with the instruction to “Relax” (duration between “Right” and “Relax”, is 5 s). The sound dynamically shifts “Right” or “Left” along the azimuth, toward the direction of the imagined movement.

## Q&A paradigm

[Example – “Right” arm versus “Left” arm *Q&A* run, where a “Right” arm imagined movement indicates a “Yes” response and a “Left” arm imagined movement indicates a “No” response]

“We will now begin a session with questions.

If you want to say “Yes” – imagine moving your “Right” arm.

If you want to say “No” – imagine moving your “Left” arm.

Try to imagine the movement as soon as the question ends and then relax.

Do not worry if you cannot answer one question – just relax and focus on the next.

Here we go.”

[Example – for a “Yes” trial in a “Right” arm versus “Left” arm *Q&A* run]

Trial begins with a “Yes” question – and ends with the instruction to “Relax” (duration between “Yes” question *End* and “Relax”, is 5 s).

Supplementary Note 2. Questions used in the Q&A task for binary Yes/No responses, adapted from the Montreal Cognitive Assessment (MOCA) ^35^.

## Biographical questions

**Answer correctly**

50.Is your name _____________________? (insert correct answer)

51.Are you a _____________________? (man or woman – insert correct answer)

52.Are you _____________________? (married or single – insert correct answer)

53.Is your hair _____________________? (insert correct colour)

54.Are your eyes _____________________? (insert correct colour)

55.Are you _____________________? (nationality – insert correct answer)

56.Are you in your _____________________? (insert correct age bracket, e.g. 30’s, 40’s etc.)

57.Is your father’s name _____________________? (insert correct name)

58.Is your mother’s name _____________________? (insert correct name)

59.Is this _____________________ voice? (e.g. your mother’s/father’s – insert correct answer)

60.Were you born in _____________________? (insert correct month)

61.Are you _____________________ handed? (right/left – insert correct answer)

**Answer incorrectly**

1.Is your name _____________________? (insert incorrect answer)

2.Are you a _____________________? (man or woman – insert incorrect answer)

3.Are you _____________________? (married or single – insert incorrect answer)

4. Is your hair _____________________? (insert incorrect colour)

5. Are your eyes _____________________? (insert incorrect colour)

6.Are you ____________? (nationality – insert incorrect answer)

7.Are you in your 90s?

8.Is your father’s name ____________? (insert incorrect name)

9.Is your mother’s name ____________? (insert incorrect name)

10.Is this the voice of _____________________? (insert name of someone other than the speaker)

11.Were you born in _____________________? (insert month other than month of birth)

12.Are you _____________________ handed? (right/left – insert incorrect answer)

## Situational

**Answer correctly**

62.Are you in a _____________________? (insert correct answer – bed or chair)

63.Are you at _____________________? (insert correct location, such as home or the hospital)

64.Are you awake?

65.Do you live in _____________________? (insert correct location)

66.Do you live in the UK?

67.Is the year _____? (insert correct year)

68.Do we live on earth?

69.Is it day time?

70.Are you indoors?

71.Are you facing the _____________________? (insert correct answer, e.g., wall, door, window)

72.Is the _____________________ off? (insert correct answer, e.g., TV, radio)

73.Are you wearing a cap?

**Answer incorrectly**

13.Are you in a _____________________? (bed or chair – insert incorrect answer)

14.Are you in _____________________? (insert incorrect location, e.g., home or hospital)

15.Are you asleep?

16.Do you live in _____________________? (insert incorrect location)

17.Do you live n _____________________? (insert incorrect country)

18.Is the year 1930?

19.Do we live on mars?

20.Is it night time?

21.Are you outside?

22.Are you facing the floor?

23.Is the _____________________ on? (insert incorrect answer, e.g., TV, radio)

24.Are you wearing a scarf?

## Basic logic

74.Is water wet?

75.Is light bright?

76.Is sunshine warm?

77.Do stones sink?

78.Are mice smaller than elephants?

79.Are babies younger than grandmas?

80.Do apples grow on trees?

81.Are there 4 seasons?

82.Is winter colder than Summer?

83.Are bananas and apples fruit?

84.Are trains and bicycles transport?

85.Do watches and rulers measure?

25.Is water dry?

26.Is light dark?

27.Is sunshine cold?

28.Do stones float?

29.Are mice bigger than elephants?

30.Are grandmas younger than babies?

31.Do apples grow on lamp posts?

32.Are there only 2 seasons?

33.Is Winter warmer than Summer?

34.Do bananas and apples measure?

35.Are trains and bicycles fruit?

36.Are watches and rulers transport?

## Numbers and letters

86.Are A-B-C-D letters?

87.Are 1-2-3-4 numbers?

88.Is 1 equal to 1?

89.Is 3 plus 1 equal to 4?

90.Is 2 minus 2 equal to 0?

91.Is A before Z in the alphabet?

92.Is Y after B in the alphabet?

93.Is 5 greater than 3?

94.Is 2 less than 6?

95.Is 50p plus 50p equal to 1 pound?

96.Is X-Y-Z, Z-Y-X backwards?

97.Is 7-4-2, 2-4-7 backwards?

37.Are 1-2-3-4 letters?

38.Are A-B-C-D numbers?

39.Is 1 equal to 2?

40.Is 3 plus 1 equal to 8?

41.Is 2 minus 2 equal to 4?

42.Is A after Z in the alphabet?

43.Is Y before B in the alphabet?

44.Is 5 greater than 6?

45.Is 2 less than 1?

46.Is 50p + 20p equal to 1 pound?

47.Is X-Y-Z, Z-P-Q backwards?

48.Is 7-4-2, 1-2-3 backwards?

Extras

98.Relax

# Supporting Information: Additional Exploratory Results

Supplementary Table 4. Summary of the accuracy of decoded responses during (a) the task period and (b) the reference period, per question category and group.

1. Accuracy metrics for the decoded responses during the task period, per question category and group.

| Question  Category | Group | Accuracy  (%) | Bias  Index | False Negative  Yes (%) | Miss Rate  No (%) |
| --- | --- | --- | --- | --- | --- |
| Biographical | UWS | 77.6 | 0.008 | 22 | 22.8 |
|  | MCS | 78 | -0.013 | 22.6 | 21.3 |
|  | LIS | 85.7 | -0.008 | 14.6 | 14 |
|  | AB | 87.2 | 0.1 | 7.8 | 17.8 |
|  |  |  |  |  |  |
| Situation | UWS | 77.2 | 0.006 | 22.5 | 23.1 |
|  | MCS | 76.9 | -0.004 | 23.3 | 22.9 |
|  | LIS | 85.7 | -0.008 | 14.7 | 13.8 |
|  | AB | 83.6 | 0.083 | 12.2 | 20.6 |
|  |  |  |  |  |  |
| Basic  Logic | UWS | 76.1 | 0.02 | 22.9 | 24.9 |
|  | MCS | 77.7 | -0.005 | 22.5 | 22 |
|  | LIS | 84.2 | -0.028 | 17.2 | 14.4 |
|  | AB | 85.3 | 0.016 | 13.9 | 15.5 |
|  |  |  |  |  |  |
| Numbers  and  Letters | UWS | 77.7 | 0.002 | 22.2 | 22.4 |
|  | MCS | 77.5 | 0.008 | 22.1 | 22.9 |
|  | LIS | 83.6 | 0.022 | 15.3 | 17.5 |
|  | AB | 89.4 | 0 | 10.6 | 10.6 |

1. Accuracy metrics for the decoded responses during the reference period, per question category and group.

| Question  Category | Group | Accuracy  (%) | Bias  Index | False Negative  Yes (%) | False Negative  No (%) |
| --- | --- | --- | --- | --- | --- |
| Biographical | UWS | 61.3 | 0.016 | 37.9 | 39.5 |
|  | MCS | 62.3 | -0.027 | 39 | 36.3 |
|  | LIS | 62.4 | -0.045 | 39.8 | 35.4 |
|  | AB | 61.3 | -0.007 | 39.1 | 38.4 |
|  |  |  |  |  |  |
| Situation | UWS | 61.7 | -0.006 | 38.6 | 38 |
|  | MCS | 60.6 | 0.042 | 37.3 | 41.5 |
|  | LIS | 60.7 | -0.004 | 39.5 | 39.2 |
|  | AB | 62.8 | -0.017 | 38.1 | 36.4 |
|  |  |  |  |  |  |
| Basic  Logic | UWS | 64.9 | -0.019 | 36 | 34.1 |
|  | MCS | 61.5 | 0.005 | 38.3 | 38.8 |
|  | LIS | 61.2 | 0.04 | 36.8 | 40.8 |
|  | AB | 61.6 | -0.018 | 39.2 | 37.5 |
|  |  |  |  |  |  |
| Numbers  and  Letters | UWS | 64.2 | 0.117 | 30 | 41.7 |
|  | MCS | 61.1 | 0.033 | 37.2 | 40.6 |
|  | LIS | 54.2 | 0.068 | 42.4 | 49.2 |
|  | AB | 65.8 | -0.261 | 47.2 | 21.1 |

Supplementary Note 3. Guidance on interpretation of results in Supplementary Tables 2 and 3.

Let *N*=*TP*+*TN*+*FP*+*FN,* where *TP*, *TN*, *FP*, and *FN* are defined in Supplementary Table 2 above. Therefore, each metric in Supplementary Table 4 (a) and (b) above is defined and calculated as follows.

**Accuracy (%):**  The percentage of correct “Yes” (TP) and “No” (TN) responses; $\frac{TP+TN}{N} \times100$

**Predicted Yes:** $TP+FP$, equal to all predictions of “Yes”.

**Predicted No:** $TN+FN$ , equal to all predictions of “No”.

**Bias Index (+1 to – 1):** The proportion of “Yes” vs “No” predictions, regardless of correctness; $\frac{\left( TP+FP \right)-(TN+FN)}{N} \times100$ , therefore, always “Yes” = +1 and always “No” = - 1, and no bias equals zero.

**False Negative “Yes” and Miss Rate “No”,** are calculated as follows.

${\%False Negative}_{Yes} =100\times\frac{FN}{TP+FN}$ = 100 x (1 – Recall *_Yes_*)

${\%Miss Rate}_{No} =100\times\frac{FP}{TN+FP}$ = 100 x (1 – Recall *_No_*)

where Recall *_Yes_* = $\frac{TP}{TP+FN}$ , and Recall *_No_* = $\frac{TN}{TN+FP}$

Supplementary Table 5. Summary of the questions with the lowest and highest ranking accuracies, for a subset of participants, one participant selected per group.

| UWS participant | | | | | |
| --- | --- | --- | --- | --- | --- |
| Low | # | Q cat | Response | Question | Ratio C |
|  | 27 | Bio | Yes | Are you in a relationship? | 0.615 |
|  | 27 | Sit | Yes | Are you wearing a cap? | 0.615 |
|  | 27 | Sit | No | Are you facing the floor? | 0.630 |
|  | 29 | Log | Yes | Is water wet? | 0.641 |
|  | 29 | Log | No | Is Winter warmer than Summer? | 0.648 |
|  |  |  |  |  |  |
| High | 27 | Sit | No | Do you live in Barcelona? | 0.904 |
|  | 27 | Bio | Yes | Is your father's name Alex? | 0.874 |
|  | 29 | Log | Yes | Is winter colder than Summer? | 0.855 |
|  | 29 | Num | Yes | Is A before Z in the alphabet? | 0.848 |
|  | 29 | Num | Yes | Is Y after B in the alphabet? | 0.848 |
|  |  |  |  |  |  |
| MCS participant | | | | | |
| Low | 54 | Num | Yes | Is 7-4-2, 2-4-7 backwards? | 0.674 |
|  | 55 | Sit | Yes | Are you indoors? | 0.676 |
|  | 54 | Num | No | Is 1 equal to 2? | 0.678 |
|  | 55 | Sit | No | Is it night time? | 0.684 |
|  | 55 | Sit | No | Is the TV on? | 0.684 |
|  |  |  |  |  |  |
| High | 57 | Bio | No | Is your father’s name Matthew? | 0.884 |
|  | 54 | Num | No | Is 5 greater than 6? | 0.874 |
|  | 57 | Log | No | Are grandmas younger than babies? | 0.860 |
|  | 54 | Num | No | Is X-Y-Z, Z-P-Q backwards? | 0.856 |
|  | 57 | Bio | Yes | Is this Heather's voice? | 0.839 |
|  |  |  |  |  |  |
| LIS participant | | | | | |
| Low | 62 | Num | No | Is 2 less than 1? | 0.765 |
|  | 62 | Num | No | Is 7-4-2, 1-2-3 backwards? | 0.768 |
|  | 60 | Sit | Yes | Are you in your chair? | 0.777 |
|  | 62 | Log | No | Are mice bigger than elephants? | 0.781 |
|  | 62 | Num | No | s A after Z in the alphabet? | 0.781 |
|  |  |  |  |  |  |
| High | 60 | Sit | No | Do you live in Paris? | 0.917 |
|  | 63 | Bio | No | Is your mother's name Louise? | 0.914 |
|  | 60 | Sit | No | Is the year 1930? | 0.910 |
|  | 62 | Num | Yes | Are 1-2-3-4 numbers? | 0.910 |
|  | 60 | Sit | No | Do you live in France? | 0.903 |
|  |  |  |  |  |  |
| AB participant | | | | | |
| Low | 3 | Sit | No | Is the year 1930? | 0.400 |
|  | 3 | Log | No | Do bananas and apples measure? | 0.467 |
|  | 3 | Bio | Yes | Are you left handed? | 0.533 |
|  | 3 | Sit | Yes | Do we live on earth? | 0.533 |
|  | 3 | Log | Yes | Do apples grow on trees? | 0.533 |
|  |  |  |  |  |  |
| High | 3 | Bio | No | Are you unmarried? | 1 |
|  | 3 | Bio | No | Is your father's name Matthew? | 1 |
|  | 3 | Bio | No | Is your mother's name Louise? | 1 |
|  | 3 | Sit | No | Do we live on mars? | 1 |
|  | 3 | Log | No | Is water dry? | 1 |

Supplementary Note 4. Description of Supplementary Table 4 headers.

**#** = the number of times the participant was presented with the corresponding question.

**Q cat** = Question category; Biographical (Bio), Situational (Sit), Basic Logic (Log), and Numbers and Letters (Num).

**Response:** The correct classification (expected response) for the corresponding question.

**Question:** The question, as it was phrased for the selected participant..

**Ratio C** = the ratio of correct responses (out of the number of times the question was presented).

Supplementary Note 5. Benchmarks.

**The Able Bodied (AB) participants served as a benchmark, to demonstrate the potential for the MI-BCI, to decode a 2-class imagined movement, when the individual has intact conscious awareness and cognitive capacity. Therefore, as can be seen here, the AB participant only had three presentations of each question, resulting in a wide range between the lowest and highest ranks.**

Supplementary Table 6. Functional Connectivity: Description of the network nodes, and the corresponding Montreal Neurological Institute (MNI) Coordinates, replicated from Aubinet *et al.* (2018).

| Networks | Nodes | Coordinates |
| --- | --- | --- |
|  |  |  |
| DMN | mPFC | x = −1 y = 54 z = 27 |
|  | PCC | x = 0 y = −52 z = 27 |
|  |  |  |
| FPN | Left dlPFC | x = −43 y = 22 z = 34 |
|  | Right dlPFC | x = 43 y = 22 z = 34 |
|  |  |  |
|  | Left IPL | x = −43.1 y = −47 z = 45.4 |
|  | Right IPL | x = 46.3 y = −47.6 z = 48.2 |
|  |  |  |
| Thalamocortical | Left Thalamus | x = −8 y = −20 z = 6 |
|  | Right Thalamus | x = 8 y = −20 z = 6 |
|  |  |  |
| Auditory | Left STG | x = −44 y = −6 z = 11 |
|  | Right STG | x = 44 y = −6 z = 11 |
|  |  |  |

**Abbreviations:** DMN = Default Mode Network; FPN = Fronto-Parietal Network; mPFC = medial PreFrontal cortex; PCC = Posterior Cingulate Cortex; dlPFC = dorsolateral Prefrontal Cortex; IPL = Inferior Parietal Lobule; STG = Superior Temporal Gyrus.

Supplementary Note 6. Functional connectivity (FC) differences were derived from Spectral Functional Connectivity Difference (SFCD) matrices based on imaginary coherence (iCOH) between twelve cortical regions across six frequency bands (see Methods). The resulting 6x66 FC matrix was thresholded at α = 0.01, with results visualised in Supplementary Figure 6. Positive and negative SFCD values indicate stronger differences between the two conditions, with positive values possibly indicating faster phase-lagged communication in the first group (e.g., for UWS v. MCS, group 1 = UWS and group 2 = MCS) and vice versa. Task-related FC differences were observed only between the UWS group and the MCS or LIS groups.

Supplementary Table 7. Task- and frequency-specific significant (α = 0.01) functional connectivity (FC) differences (imaginary coherence, iCOH) between cortical nodes across diagnostic groups. Comparisons (UWS vs. MCS, UWS vs. LIS, and MCS vs. LIS) are shown by paradigm (Assessment, Training, Q&A) and frequency band, and iCOH (values quantify the strength of phase coupling between the nodes, regardless of the sign (-/+).

| Comparison | Paradigm | Freq-band | Node A | Net | Node B | Net | iCOH val |
| --- | --- | --- | --- | --- | --- | --- | --- |
|  |  |  |  |  |  |  |  |
| UWS versus MCS | Assessment | Alpha | PCC | DMN | rdlPFC | FPN | 0.184 |
|  | Assessment | Alpha | rdlPFC | FPN | rIPL | FPN | -0.214 |
|  | Assessment | Alpha | rdlPFC | FPN | lThal | Thal. | 0.186 |
|  | Q&A | Alpha | PCC | DMN | rdlPFC | FPN | 0.163 |
|  | Q&A | Alpha | MPFC | DMN | rIPL | FPN | 0.187 |
|  | Q&A | Alpha | rdlPFC | FPN | rIPL | FPN | -0.182 |
|  | Q&A | Alpha | MPFC | DMN | lThal | Thal. | 0.154 |
|  | Q&A | Alpha | rdlPFC | FPN | lThal | Thal. | -0.168 |
|  | Q&A | Alpha | MPFC | DMN | rThal | Thal. | 0.19 |
|  | Q&A | Alpha | rdlPFC | FPN | rThal | Thal. | -0.196 |
|  | Q&A | Alpha | MPFC | DMN | rSTG | Aud. | 0.184 |
|  | Q&A | Alpha | rdlPFC | FPN | rSTG | Aud. | -0.226 |
|  |  |  |  |  |  |  |  |
| UWS versus LIS | Assessment | Alpha | MPFC | DMN | rIPL | FPN | 0.216 |
|  | Assessment | Alpha | rdlPFC | FPN | rIPL | FPN | -0.204 |
|  | Training | Alpha | MPFC | DMN | rIPL | FPN | 0.105 |
|  | Training | Alpha | rdlPFC | FPN | rSTG | Aud. | 0.117 |
|  | Q&A | Theta | rdlPFC | FPN | lThal | Thal. | -0.172 |
|  | Q&A | Theta | rdlPFC | FPN | rThal | Thal. | -0.158 |
|  | Q&A | Theta | rdlPFC | FPN | rSTG | Aud. | -0.159 |
|  | Q&A | Alpha | MPFC | DMN | PCC | DMN | 0.169 |
|  | Q&A | Alpha | PCC | DMN | rdlPFC | FPN | 0.157 |
|  | Q&A | Alpha | MPFC | DMN | rIPL | FPN | 0.173 |
|  | Q&A | Alpha | rdlPFC | FPN | rIPL | FPN | -0.215 |
|  | Q&A | Alpha | MPFC | DMN | lThal | Thal. | 0.188 |
|  | Q&A | Alpha | rdlPFC | FPN | lThal | Thal. | -0.155 |
|  | Q&A | Alpha | rIPL | FPN | lThal | Thal. | 0.167 |
|  | Q&A | Alpha | MPFC | DMN | rThal | Thal. | 0.215 |
|  | Q&A | Alpha | rdlPFC | FPN | rThal | Thal. | -0.196 |
|  | Q&A | Alpha | MPFC | DMN | rSTG | Aud. | 0.208 |
|  | Q&A | Alpha | rdlPFC | FPN | rSTG | Aud. | -0.196 |
|  |  |  |  |  |  |  |  |
| MCS versus LIS |  |  |  |  |  |  |  |
|  | Training | Alpha | MPFC | DMN | lIPL | FPN | -0.21 |
|  | Training | Alpha | MPFC | DMN | rSTG | Aud. | 0.198 |
|  |  |  |  |  |  |  |  |

**Abbreviations:** DMN = Default Mode Network; FPN = Fronto-Parietal Network; mPFC = medial PreFrontal cortex; PCC = Posterior Cingulate Cortex; dlPFC = dorsolateral Prefrontal Cortex; IPL = Inferior Parietal Lobule; STG = Superior Temporal Gyrus. Where there is an ‘r’ or ‘l’ prefix, this denoted ‘right’ or ‘left’ respectively. Freq-band refers to the frequency-band (Theta = 4-7 Hz; Alpha = 8-12 Hz). Node A and Node B denote the Regions of Interest (ROIs) that form the connection (edge) in which group-level functional connectivity differences were significant (α = 0.01).


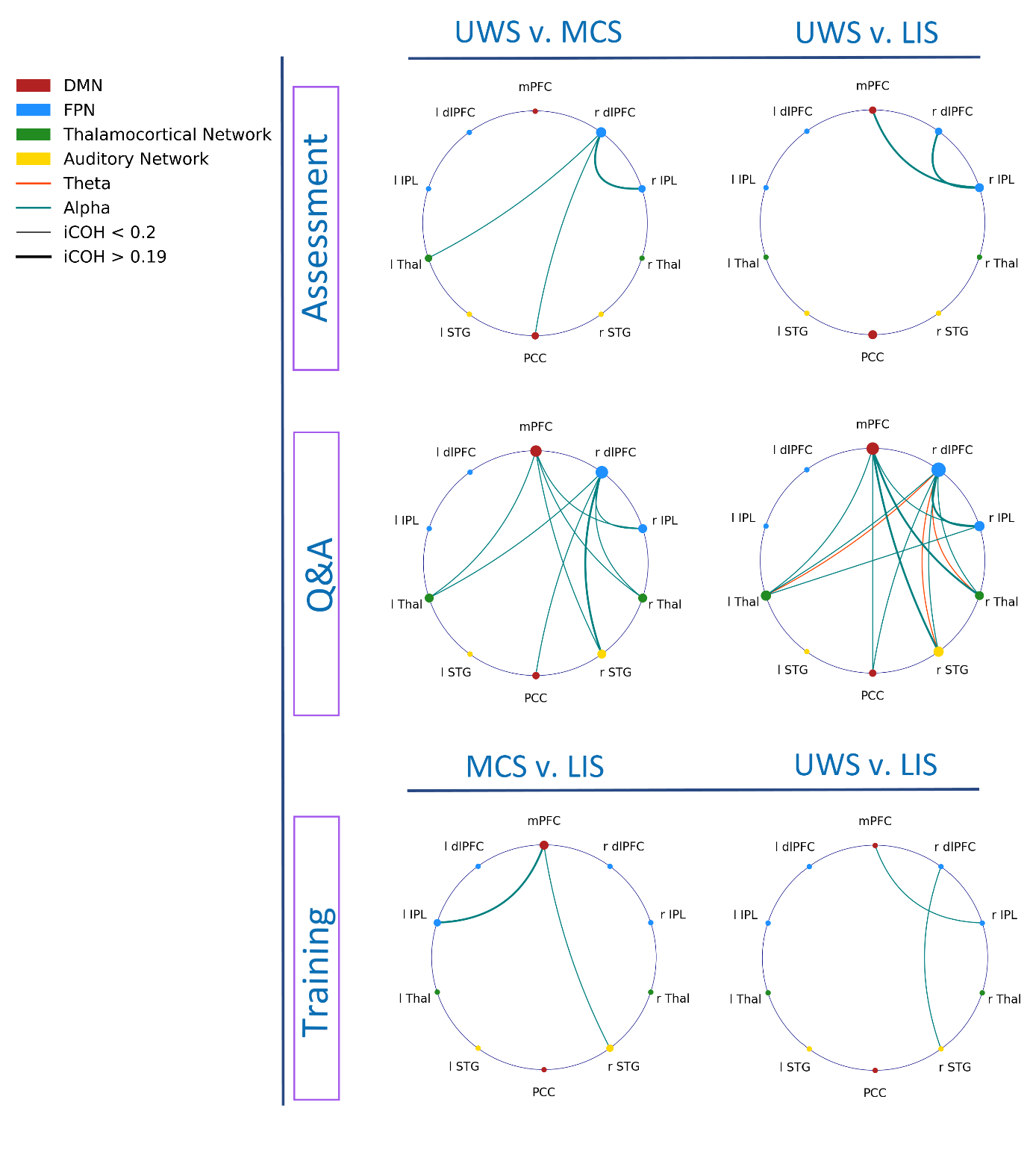


Supplementary Figure 7. Group-level differences in functional connectivity (FC) between patient groups across task paradigms. Rows correspond to the Assessment, Q&A, and Training paradigms. In Column 1, contrasts are shown for UWS vs. MCS in the Assessment and Q&A paradigms, and for MCS vs. LIS in Training. Column 2 displays contrasts for UWS vs. LIS across all three paradigms. Each circular graph depicts source-level FC contrasts between cortical network nodes: default mode network (DMN, red; mPFC, PCC), frontoparietal network (FPN, blue; l/r dlPFC, l/r IPL), thalamocortical network (green; l/r Thal), and auditory network (yellow; l/r STG). Node size reflects the number of significant connections. Edges represent statistically significant group differences (α = 0.01) in imaginary coherence (iCOH) within the alpha (green) and theta (orange) frequency bands. Statistical significance was determined using a non-parametric max-statistics approach with Monte Carlo permutation testing (1000 replications) to control for multiple comparisons. Thicker lines denote stronger phase-lagged connectivity ^1^ (iCOH > 0.1).

Supplementary Note 7. Results of the exploratory Functional Connectivity Analysis.

***Assessment paradigm FC*:** During the assessment task, both UWS-MCS and UWS-LIS comparisons revealed FC differences involving the right dorsolateral prefrontal cortex (r-dlPFC). Specifically, UWS-MCS differences were observed in its connections with the left thalamus (l-Thal) and the right inferior parietal lobule (r-IPL), while UWS-LIS differences occur in right dlPFC-right IPL connectivity. These findings are consistent with thalamo-frontal disruptions in UWS ^2–5^. Additionally, the UWS-LIS FC differences found between the mPFC and the right IPL, indicate disrupted frontoparietal and DMN-FPN interactions that may reflect reduced attentional focus in UWS ^6^.

***Training paradigm FC*:** In the training task, FC differences were observed between the r-STG (auditory cortex) and the mPFC in the MCS-LIS comparison, and between the r-STG and the r-dlPFC in the UWS-LIS comparison. These results point to emerging disruption of auditory network interactions, particularly with frontal and DMN, consistent with prior findings in MCS patients compared with healthy controls ^7^. Again altered mPFC and r-IPL connectivity was observed in the UWS-LIS comparison, indicating reduced attentional focus in UWS ^6^.

***Q&A paradigm FC*:** The Q&A task elicited the most extensive FC differences, particularly involving the mPFC (DMN), r-dlPFC (FPN), Thalamus, and r-STG (auditory network). Differences in connectivity between the PCC and dlPFC suggest impaired cognitive control and situational awareness in UWS. These patterns indicate both thalamo-frontal disconnection and disrupted auditory processing during cognitively demanding tasks in this group.

***General FC findings*:** Across paradigms, the nodes most frequently involved in significant group differences were the mPFC and PCC (DMN), r-dlPFC and r-IPL (FPN), bilateral thalamus (Thalamocortical), and r-STG (Auditory). In healthy individuals, the mPFC supports self-referential decision-making ^8^, while the PCC – an integrative DMN hub with dense frontoparietal connections ^9^ – monitors internal and external environments ^10^. In patients with disorders of consciousness (DoC), the typical deactivation of the DMN (necessary to engage executive control networks during tasks) is reduced in MCS and absent in UWS ^11^.

The dlPFC, a key FPN node, governs executive functions like cognitive switching, inhibition, and verbal fluency ^12^. Stimulation of the dlPFC via rTMS ^13–15^ or tDCS ^16,17^ has shown therapeutic potential, particularly for MCS patients. The thalamus is central to forebrain integration and arousal regulation – both essential to consciousness ^18–20^. Although UWS patients can exhibit primary auditory cortex activation in response to external stimuli ^21^, they often lack connectivity with higher-order auditory areas, a pattern more preserved in MCS ^7,22^ . In the present training paradigm, however, disruptions of the auditory network were also evident in MCS patients when compared with the LIS group. Notably, auditory-FPN connectivity has been linked to conscious auditory perception ^23,24^. In line with this, Demertzi et al. (2014) found that DMN and auditory network features provided 85.3% accuracy in classifying PDoC patients from healthy controls using fMRI-based machine learning ^25^.

All significant group differences (α = 0.01) were found in the alpha band (8-12 Hz), extending to the theta band (4-7 Hz) for the UWS-LIS comparisons in the Q&A paradigm. FC differences were not observed during the Feedback paradigm, and differences between the MCS and LIS groups were only observed in the comparisons for the Q&A paradigm. Complete statistical results are reported in Supplementary Table 7.

Although exploratory, this FC analysis revealed key network-level differences between UWS and both MCS and LIS, across Assessment, Training, and Q&A paradigms, involving networks that subserve consciousness; the Default Mode Network (DMN), Fronto-Parietal Network (FPN), Thalamocortical, and Auditory networks. These group differences centred on thalamo-frontal alterations in UWS, aligning with known mechanisms of impaired awareness in DoC ^5,26,27^. Down regulation of corticothalamic neural activity has been recognised as a pathophysiological mechanism underlying disorders of consciousness ^5^. Thalamo-frontal connectivity, more specifically, has been demonstrated to be necessary for task-related (goal-directed) cognitive processes in a PDoC cohort, with differences in the modulation of thalamo-frontal connectivity distinguishing ‘responders’ from ‘non-responders’ (as opposed to UWS from MCS) on two tasks (passive listening and mental counting) ^28^. Disruptions in mPFC-IPL connectivity were consistently observed in UWS comparisons across the assessment, training, and Q&A paradigms, and also in the MCS-LIS comparison during the training paradigm – indicating reduced attentional focus of the UWS group in particular ^6^.

Importantly, this analysis examined FC during active MI task engagement, demonstrating that MI-related FC differences can be diagnostically informative, revealing differences in context-specific neural patterns. In particular, thalamo-frontal alterations in UWS were consistently observed during cognitively demanding tasks, particularly in comparison to MCS and LIS in the Q&A paradigm. These findings support the hypothesis that dynamic, goal-directed neural processing is more precisely captured through task-based protocols.

Two key observations of the FC analysis centre on the absence of FC differences. Firstly, the absence of a feedback task-related FC difference is interesting. The feedback paradigm is unique in presenting an auditory stimulus during the task period: pink noise or music was delivered binaurally via headphones, with the sound dynamically shifting along the azimuth toward the direction of the imagined movement, as determined by the classifier. A study by Laureys et al. (2004), demonstrated the activation of the primary auditory cortex in UWS patients by exogenous stimuli, despite disruptions to functional connections with higher-order associative areas ^2,29^. This preserved bottom-up sensory processing may mask deficits in top-down cognitive control that are observed in the task-related FC during the assessment, training and Q&A paradigms where no audio feedback is present during the motor imagery task period. Secondly, is the observed absence of a difference in task-related FC between the MCS and LIS groups, for the feedback and Q&A paradigm comparisons. However, research has demonstrated preservation of frontoparietal and higher-order cortices in the MCS compared to the UWS populations ^7,30^ – and equally, cognitive impairments are also not uncommon in the LIS population ^31,32^. In this context, the consistent involvement of the alpha frequency band in the observed network connectivity aligns with prior evidence that alpha-band network efficiency correlates with behavioural awareness as indexed by CRS-R scores ^33^. This is consistent with findings that reduced alpha power is linked to diffuse cortical damage in post-anoxic patients ^34^.

Importantly, this analysis examined FC during active MI task engagement, demonstrating that MI-related FC differences can be diagnostically informative, revealing differences in context-specific neural patterns. In particular, thalamo-frontal alterations in UWS were consistently observed during cognitively demanding tasks, particularly in comparison to MCS and LIS in the Q&A paradigm. These findings support the hypothesis that dynamic, goal-directed neural processing is more precisely captured through task-based protocols.

# Supporting Information: Description of the Supplementary Data workbook

Supplementary Note 8. Description of the Supplementary Data workbook.

The accompanying *Supplementary Data* Excel workbook, contains the numerical source data, descriptive statistics, and numbers of units of analysis underlying figures presenting empirical results in the main manuscript. The workbook comprises nine sheets: Supplementary Data A, source data for Figure 4; Supplementary Data B, source data for Figure 5; Supplementary Data C, source data for Figure 6; Supplementary Data D, source data for Figure 7; Supplementary Data E, source data for Figure 8; Supplementary Data F, statistical analysis of regional CSP–MuI weights corresponding to Figure 9; Supplementary Data G, descriptive statistics for regional CSP–MuI weights corresponding to Figure 9; Supplementary Data H, numbers of units of analysis contributing to the Figure 9 regional analyses; and Supplementary Data I, source data for the functional connectivity results shown in Supplementary Figure 7.

# Supporting Information References

1. Nolte, G. *et al.* Identifying true brain interaction from EEG data using the imaginary part of coherency. *Clin. Neurophysiol.* **115**, 2292–2307 (2004).

2. Laureys, S., Owen, A. M. & Schiff, N. D. Brain function in coma, vegetative state, and related disorders. *Lancet Neurol.* **3**, 537–546 (2004).

3. Monti, M. M. Cognition in the vegetative state. *Annu. Rev. Clin. Psychol.* **8**, (2012).

4. Demertzi, A., Soddu, A. & Laureys, S. Consciousness supporting networks. *Curr. Opin. Neurobiol.* **23**, 239–244 (2013).

5. Edlow, B. L., Claassen, J., Schiff, N. D. & Greer, D. M. Recovery from disorders of consciousness: mechanisms, prognosis and emerging therapies. *Nat. Rev. Neurol.* **17**, 135–156 (2021).

6. Stawarczyk, D., Majerus, S., Maquet, P. & D’Argembeau, A. Neural correlates of ongoing conscious experience: Both task-unrelatedness and stimulus-independence are related to default network activity. *PLoS One* **6**, (2011).

7. Boly, M. *et al.* Auditory Processing in Severely Brain Injured Patients: Differences between the Minimally Conscious State and the Persistent Vegetative State. *Arch. Neurol.* **61**, 233–238 (2004).

8. Yasin, S. *et al.* Self-Enhancement and the Medial Prefrontal Cortex: The Convergence of Clinical and Experimental Findings. *Brain Sci.* **12**, (2022).

9. Leech, R., Braga, R. & Sharp, D. J. Echoes of the brain within the posterior cingulate cortex. *J. Neurosci.* **32**, 215–222 (2012).

10. Raichle, M. E. *et al.* A default mode of brain function. *Proc. Natl. Acad. Sci. U. S. A.* **98**, 676–682 (2001).

11. Crone, J. S. *et al.* Deactivation of the default mode network as a marker of impaired consciousness: An fmri study. *PLoS One* **6**, (2011).

12. Panikratova, Y. R. *et al.* Functional connectivity of the dorsolateral prefrontal cortex contributes to different components of executive functions. *Int. J. Psychophysiol.* **151**, 70–79 (2020).

13. Naro, A. *et al.* A Single Session of Repetitive Transcranial Magnetic Stimulation Over the Dorsolateral Prefrontal Cortex in Patients With Unresponsive Wakefulness Syndrome: Preliminary Results. *Neurorehabil. Neural Repair* **29**, 603–613 (2015).

14. Xia, X. *et al.* Long-lasting repetitive transcranial magnetic stimulation modulates electroencephalography oscillation in patients with disorders of consciousness. *Neuroreport* **28**, 1022–1029 (2017).

15. Fan, J., Zhong, Y., Wang, H., Aierken, N. & He, R. Repetitive transcranial magnetic stimulation improves consciousness in some patients with disorders of consciousness. *Clin. Rehabil.* **36**, 916–925 (2022).

16. Bai, Y. *et al.* TDCS modulates cortical excitability in patients with disorders of consciousness. *NeuroImage Clin.* **15**, 702–709 (2017).

17. Martens, G. *et al.* Behavioral and electrophysiological effects of network-based frontoparietal tDCS in patients with severe brain injury: A randomized controlled trial. *NeuroImage Clin.* **28**, 102426 (2020).

18. Schiff, N. D. Central thalamic contributions to arousal regulation and neurological disorders of consciousness. *Ann. N. Y. Acad. Sci.* **1129**, 105–118 (2008).

19. Schiff, N. D. Recovery of consciousness after brain injury: a mesocircuit hypothesis. *Trends Neurosci.* **33**, 1–9 (2010).

20. Shah, S. A. *et al.* Executive attention deficits after traumatic brain injury reflect impaired recruitment of resources. *NeuroImage Clin.* **14**, 233–241 (2017).

21. Laureys, S. *et al.* Auditory processing in the vegetative state. *Brain* **123**, 1589–1601 (2000).

22. Aubinet, C. *et al.* Clinical subcategorization of minimally conscious state according to resting functional connectivity. *Hum. Brain Mapp.* **39**, 4519–4532 (2018).

23. Hasson, U., Skipper, J. I., Nusbaum, H. C. & Small, S. L. Abstract Coding of Audiovisual Speech: Beyond Sensory Representation. *Neuron* **56**, 1116–1126 (2007).

24. Dehaene, S. & Changeux, J. P. Experimental and Theoretical Approaches to Conscious Processing. *Neuron* **70**, 200–227 (2011).

25. Demertzi, A. *et al.* Multiple fMRI system-level baseline connectivity is disrupted in patients with consciousness alterations. *Cortex* **52**, 35–46 (2014).

26. Cacciola, A. *et al.* Functional brain network topology discriminates between patients with minimally conscious state and unresponsivewakefulness syndrome. *J. Clin. Med.* **8**, (2019).

27. Enciso-Olivera, C. O. *et al.* Structural and functional connectivity of the ascending arousal network for prediction of outcome in patients with acute disorders of consciousness. *Sci. Rep.* **11**, 1–12 (2021).

28. Monti, M. M. *et al.* Thalamo-frontal connectivity mediates top-down cognitive functions in disorders of consciousness. *Neurology* **84**, 167–173 (2015).

29. Laureys, S. *et al.* Brain function in the vegetative state. *Adv. Exp. Med. Biol.* **550**, 229–238 (2004).

30. Gosseries, O., Di, H., Laureys, S. & Boly, M. Measuring Consciousness in Severely Damaged Brains. *Annu. Rev. Neurosci.* **37**, 457–478 (2014).

31. New, P. W. & Thomas, S. J. Cognitive impairments in the locked-in syndrome: A case report. *Arch. Phys. Med. Rehabil.* **86**, 338–343 (2005).

32. Rousseaux, M., Castelnot, E., Rigaux, P., Kozlowski, O. & Danzé, F. Evidence of persisting cognitive impairment in a case series of patients with locked-in syndrome. *J. Neurol. Neurosurg. Psychiatry* **80**, 166–170 (2009).

33. Chennu, S. *et al.* Spectral Signatures of Reorganised Brain Networks in Disorders of Consciousness. *PLoS Comput. Biol.* **10**, (2014).

34. Colombo, M. A. *et al.* Beyond alpha power: EEG spatial and spectral gradients robustly stratify disorders of consciousness. *Cereb. Cortex* **33**, 7193–7210 (2023).

35. De Guise, E. *et al.* The montreal cognitive assessment in persons with traumatic brain injury. *Appl. Neuropsychol.* **21**, 128–135 (2014).
